# The Dedicated Chaperone Acl4 Escorts Ribosomal Protein Rpl4 to Its Nuclear Pre-60S Assembly Site

**DOI:** 10.1371/journal.pgen.1005565

**Published:** 2015-10-08

**Authors:** Benjamin Pillet, Juan J. García-Gómez, Patrick Pausch, Laurent Falquet, Gert Bange, Jesús de la Cruz, Dieter Kressler

**Affiliations:** 1 Unit of Biochemistry, Department of Biology, University of Fribourg, Fribourg, Switzerland; 2 Instituto de Biomedicina de Sevilla, Hospital Universitario Virgen del Rocío/CSIC/Universidad de Sevilla, Seville, Spain; 3 Departamento de Genética, Universidad de Sevilla, Seville, Spain; 4 LOEWE Center for Synthetic Microbiology and Department of Chemistry, Philipps-University Marburg, Marburg, Germany; 5 Swiss Institute of Bioinformatics, University of Fribourg, Fribourg, Switzerland; University of Edinburgh, United Kingdom

## Abstract

Ribosomes are the highly complex macromolecular assemblies dedicated to the synthesis of all cellular proteins from mRNA templates. The main principles underlying the making of ribosomes are conserved across eukaryotic organisms and this process has been studied in most detail in the yeast *Saccharomyces cerevisiae*. Yeast ribosomes are composed of four ribosomal RNAs (rRNAs) and 79 ribosomal proteins (r-proteins). Most r-proteins need to be transported from the cytoplasm to the nucleus where they get incorporated into the evolving pre-ribosomal particles. Due to the high abundance and difficult physicochemical properties of r-proteins, their correct folding and fail-safe targeting to the assembly site depends largely on general, as well as highly specialized, chaperone and transport systems. Many r-proteins contain universally conserved or eukaryote-specific internal loops and/or terminal extensions, which were shown to mediate their nuclear targeting and association with dedicated chaperones in a growing number of cases. The 60S r-protein Rpl4 is particularly interesting since it harbours a conserved long internal loop and a prominent C-terminal eukaryote-specific extension. Here we show that both the long internal loop and the C-terminal eukaryote-specific extension are strictly required for the functionality of Rpl4. While Rpl4 contains at least five distinct nuclear localization signals (NLS), the C-terminal part of the long internal loop associates with a specific binding partner, termed Acl4. Absence of Acl4 confers a severe slow-growth phenotype and a deficiency in the production of 60S subunits. Genetic and biochemical evidence indicates that Acl4 can be considered as a dedicated chaperone of Rpl4. Notably, Acl4 localizes to both the cytoplasm and nucleus and it has the capacity to capture nascent Rpl4 in a co-translational manner. Taken together, our findings indicate that the dedicated chaperone Acl4 accompanies Rpl4 from the cytoplasm to its pre-60S assembly site in the nucleus.

## Author Summary

Ribosomes are the molecular machines that generate proteins from mRNA templates. The biogenesis of eukaryotic ribosomes is an outstandingly complex process, in which around 80 ribosomal proteins and four ribosomal RNAs are accurately pieced together. Actively growing yeast cells must produce more than 160’000 ribosomal proteins per minute in order to meet the cellular demand for new ribosomes. Many ribosomal proteins are prone to aggregation and need therefore to be protected on their path from the cytoplasm to their mostly nuclear incorporation sites within ribosome precursors. Recent evidence has highlighted that specific binding partners, referred to as dedicated chaperones, may ensure the soluble expression, nuclear import and/or correct assembly of ribosomal proteins. Here, we have identified such a dedicated chaperone, termed Acl4, which exclusively interacts with and accompanies the ribosomal protein Rpl4 to its nuclear assembly site. Notably, Acl4 has the capacity to recognize Rpl4 as it is synthesized by the ribosome. Our findings emphasize that co-translational capturing of ribosomal proteins by dedicated chaperones is an advantageous strategy to provide sufficient amounts of assembly-competent ribosomal proteins. A detailed knowledge of eukaryotic ribosome assembly is instrumental to eventually understand and treat ribosomopathies, diseases frequently caused by altered functionalities of ribosomal proteins.

## Introduction

The biogenesis of ribosomes is a fundamental cellular process whose main principles are conserved from the lower eukaryote *Saccharomyces cerevisiae* to mammalian organisms. *S*. *cerevisiae* 80S ribosomes are composed of two unequal subunits, a small 40S (SSU) and a large 60S (LSU) ribosomal subunit (r-subunit), which contain 33 ribosomal protein (r-proteins) and the 18S ribosomal RNA (rRNA) or 46 r-proteins and the 25S, 5.8S, and 5S rRNA, respectively [1]. The synthesis of ribosomes basically consists in the ordered assembly of the r-proteins with the rRNAs; however, the efficient and accurate assembly of r-subunits vitally depends on a multitude (>200) of transiently acting biogenesis factors [2,3,4,5]. Ribosome assembly takes successively place in the nucleolus, nucleoplasm, and cytoplasm. Within the nucleolus, it is initiated by the transcription of the ribosomal DNA (rDNA) into a long, polycistronic 35S precursor rRNA (pre-rRNA), which contains the 18S, 5.8S, and 25S rRNAs, and a pre-5S rRNA [6]. Concomitant to transcription, the 35S pre-rRNA already associates with several SSU r-proteins and early-associating biogenesis factors to give birth to the first detectable pre-ribosomal particle, referred to as 90S or SSU-processome [2,3,4,5]. In a predominantly co-transcriptional process, the pre-rRNA within this initial pre-ribosomal particle undergoes cleavage at processing site A_2_ (for a pre-rRNA processing scheme, see S1 Fig) [7,8], thus leading to the formation of nuclear 43S pre-ribosomal particles containing the 20S pre-rRNA. These pre-40S ribosomes are rapidly exported to the cytoplasm, where they are converted in a series of concerted events, including processing of 20S pre-rRNA to 18S rRNA, into mature 40S subunits [2,3,4,5,9,10]. The first pre-60S ribosome, termed 66S particle, also assembles on nascent pre-rRNA and contains, upon termination of transcription, the 27SA_2_ pre-rRNA [2,8]. This 66S pre-ribosomal particle is associated with selected r-proteins, mainly binding to domains I and II of 25S rRNA, that promote the initial compaction of the emerging LSU and a characteristic set of early-acting biogenesis factors [2,3,11]. Maturation of and pre-rRNA processing within nuclear pre-60S particles proceeds in a hierarchical manner and involves the sequential recruitment of r-proteins, which shape and stabilize the pre-60S subunit with the aid of specific biogenesis factors. Affinity purifications have revealed the protein and pre-rRNA/rRNA composition of pre-60S particles and, accordingly, the existence of several distinct nuclear intermediates could be established [2,3,4,5,12]. Pre-60S subunits acquire export competence by associating with several export-promoting factors; at this stage, the particles contain most r-proteins and only relatively few biogenesis factors [2,3,4,13]. Upon appearance in the cytoplasm, a cascade of sequential events leads to the dissociation of the export and biogenesis factors and the incorporation of the final eight 60S r-proteins; thus enabling subunit joining and engagement of 80S ribosomes in translation [2,3,4,5,10].

An exponentially growing yeast cell must initially synthesize at least 2’000 molecules of each r-protein per minute to meet the demand for assembly-competent r-proteins [14]. Many r-proteins exhibit rather unique folds and they often contain, besides featuring in many cases a globular domain, disordered loops and extensions that stabilize the tertiary structure of rRNA [1,15,16]. Notably, eukaryotic r-subunits have acquired, when compared to their bacterial counterparts, additional rRNA elements, referred to as expansion segments (ES), and 46 eukaryote-specific r-proteins [1,15]. Moreover, many of the evolutionarily conserved r-proteins contain eukaryote-specific extensions, which, together with the ES and the eukaryote-specific r-proteins, form most of the solvent-side surface of eukaryotic ribosomes [15,17]. Conversely, the evolutionarily conserved loops and extensions mostly penetrate into the interior of the rRNA cores of the r-subunits [15,16]. In agreement with their prevalent role in mediating interactions with the negatively charged rRNA phosphate backbones, most r-proteins are very basic with their loops and extensions being especially rich in lysine and arginine residues [16]. Since ribosome assembly mainly occurs in the nucle(ol)us, most r-proteins have to undertake the cumbersome journey from their cytoplasmic site of synthesis to the nucleus. However, due to their structural characteristics and highly basic nature, they need, prior to their ribosome incorporation, to be protected from engaging in illicit interactions with non-cognate RNAs or polyanions that may promote their aggregation [18]. Despite their small size, nuclear import of r-proteins largely depends on active transport across the nuclear pore complex (NPC) [19,20,21]. Notably, importins may not only act as transporter receptors for r-proteins, but as they were shown to prevent their aggregation, a chaperone role for importins has been put forward [18].

As already implied above, the equimolar synthesis of assembly-competent r-proteins represents a major challenge for the cell. Each non-assembled r-protein likely exhibits a distinct intrinsic stability and propensity for aggregation, as suggested by the occurrence of different folds and the unequal partitioning into globular domains and disordered extensions [15]. Moreover, proper folding of a large number of, but apparently not all, r-proteins especially depends on two functionally collaborating ribosome-associated chaperone systems [22], consisting of the chaperone triad SSB/ribosome-associated complex (RAC) and the nascent polypeptide-associated complex (NAC) [23]. Since r-proteins associate at different spatiotemporal entry points with the evolving pre-ribosomal subunits, their non-assembled forms, which may in many cases be unstable [24,25], are exposed for different durations to the hostile intracellular environment before being finally stabilized by encountering the cognate rRNA binding context at the pre-ribosome. Therefore, it is not surprising that additional mechanisms, besides the general chaperone and transport systems, evolved to ensure the stable expression of r-proteins and the subsequent delivery to their assembly site.

While one such strategy, utilized by two r-proteins, comprises the initial synthesis as a precursor protein carrying an N-terminal ubiquitin moiety [26,27,28,29], an emerging and prevalent theme involves the association of r-proteins with specific binding partners, also referred to as dedicated chaperones. Recent evidence revealed that such binding partners prevent r-proteins from aggregation, promote their nuclear import and/or coordinate their assembly into pre-ribosomal particles. The ankyrin-repeat protein Yar1 interacts specifically with Rps3 (uS3 according to the recently proposed r-protein nomenclature [30]) and acts as an anti-aggregation chaperone that may accompany Rps3 to its nuclear assembly site [31,32]. The nuclear Tsr2 promotes the safe transfer of importin-bound Rps26 (eS26) to the 90S pre-ribosome [33]. The transport adaptor Syo1 binds simultaneously to Rpl5 (uL18) and Rpl11 (uL5) and mediates, upon nuclear import of the trimeric complex *via* the transport receptor Kap104, their synchronized delivery to the 5S rRNA by serving as a 5S RNP assembly platform [19,34,35]. Additionally, the eight-bladed WD-repeat β-propeller protein Sqt1 and the predicted WD-repeat β-propeller protein Rrb1 are dedicated chaperones of Rpl10 (uL16) and Rpl3 (uL3), respectively [36,37,38,39,40]. While Syo1, Sqt1, and Rrb1 recognize the N-terminal extensions of their binding partners [35,38], Yar1 mainly binds to the solvent-exposed side of the first α-helix within the N-terminal globular domain of Rps3 [31]. Interestingly, and in line with a protective function, these four chaperones were recently shown to have the capacity to capture their r-protein clients at the earliest possible moment in a co-translational manner [38].

**Fig 1:**
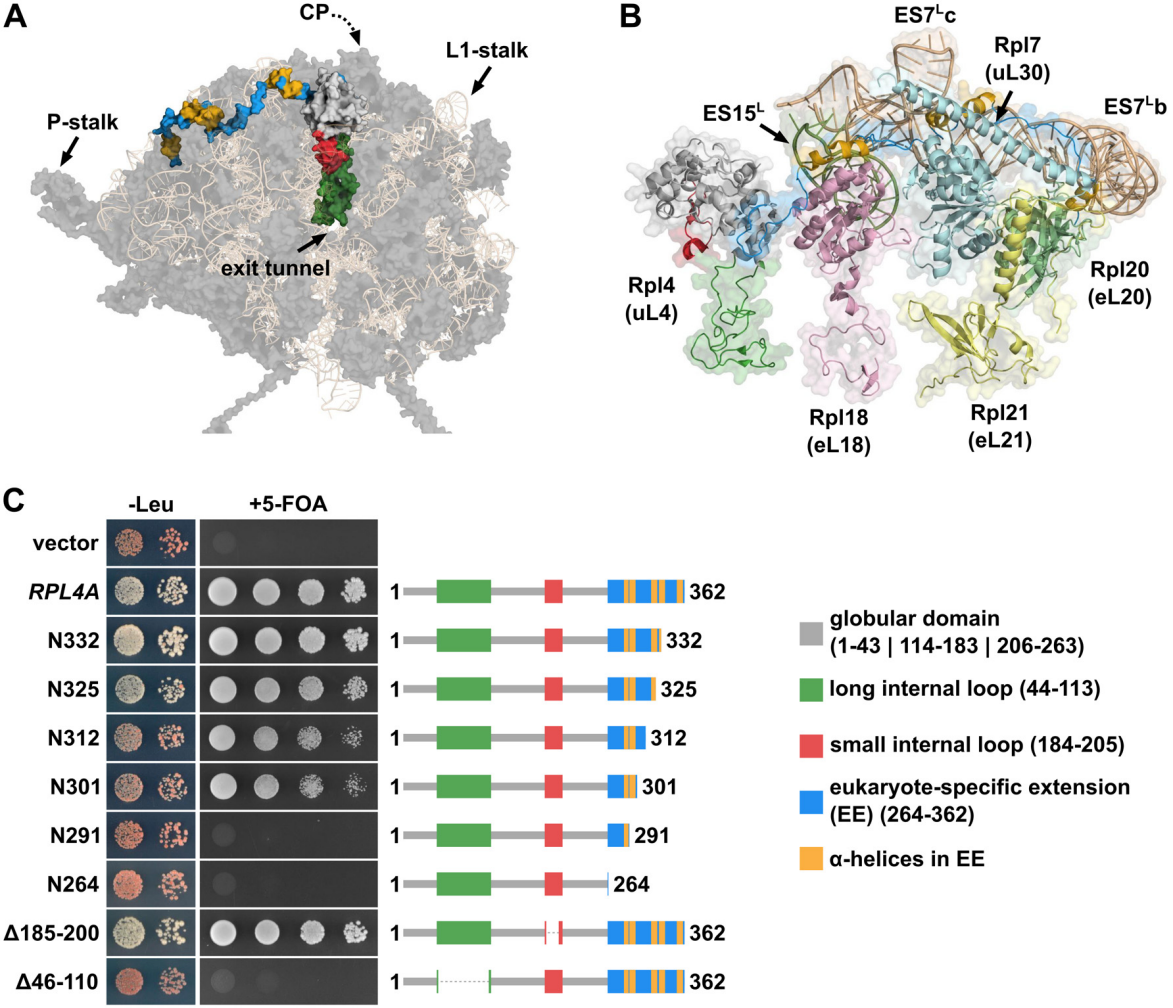
The eukaryote-specific C-terminal extension of Rpl4 is required for growth. **A,** Location of Rpl4 on mature 60S subunits. The 60S subunit is shown in its solvent-side view and has been turned along the x-axis to better visualize Rpl4. For orientation purposes, the positions of the following 60S subunit landmarks are indicated: L1-stalk, P-stalk, and polypeptide exit tunnel. Note that the central protuberance (CP) is not clearly visible due to the turned orientation of the 60S subunit. Rpl4 is shown in surface representation with the globular domain in light gray, the long internal loop in green, the small internal loop in red, the C-terminal eukaryote-specific extension in blue, and the α-helices within the eukaryote-specific extension in yellow. The rRNA components are shown in wheat as semi-transparent cartoon representation and the remaining r-proteins in dark gray as semi-transparent surface representation. The shown structure was generated in PyMOL using the PDB coordinates of the mature *S*. *cerevisiae* 60S subunit (PDB 3U5H and 3U5I; [15]). **B,** Close-up view highlighting the 60S subunit environment of the C-terminal eukaryote-specific extension of Rpl4. The different features of Rpl4 are coloured as in Fig 1A and shown in a mixed cartoon, semi-transparent surface representation. R-proteins Rpl18 (eL18), Rpl7 (uL30), Rpl20 (eL20), and Rpl21 (eL21) are coloured in pink, pale cyan, pale green, and pale yellow, respectively, and shown in a mixed cartoon, semi-transparent surface representation. The rRNA expansion segments ES15^L^ and ES7^L^ are coloured in smudge green and wheat, respectively, and shown in a mixed cartoon, semi-transparent surface representation. The position of helices ES7^L^b and ES7^L^c within ES7^L^ is indicated. **C,** *In vivo* phenotypes of cells expressing C-terminally truncated Rpl4a proteins. Empty vector (YCplac111) and plasmid-borne wild-type *RPL4A* or the indicated *rpl4a* deletion mutants, expressed under the control of the cognate promoter, were transformed into the *RPL4* shuffle strain YBP15. Transformants were restreaked on synthetic complete medium lacking leucine (SC-Leu) and cells were then spotted in 10-fold serial dilution steps onto SC-Leu (-Leu) and 5-Fluoroorotic Acid containing (+5-FOA) plates, which were incubated for 3 d at 30°C. Rpl4a is schematically depicted to highlight its features and to visually indicate the missing parts within the different Rpl4a variants.

We are interested in unravelling the assembly paths, from stable cytoplasmic synthesis, along nuclear import to ribosome incorporation, of r-proteins and in understanding if and how dedicated chaperones contribute to these events. The essential 60S r-protein Rpl4 (uL4) is a particularly interesting candidate for studying its assembly path, since it associates very early with pre-60S particles and displays remarkable structural features [2,15,41,42,43]. Rpl4 is mostly located on the solvent-side surface of the mature 60S r-subunit and is composed of a universally conserved globular domain and a prominent eukaryote-specific C-terminal extension (see Fig 1) [15,41]. Notably, the globular domain contains an insertion of a long internal loop, which penetrates deep into the interior of the 60S core and whose tip region forms part of the constriction point within the polypeptide exit tunnel [15,41]. Additionally, a small internal loop also emanates from the surface-exposed globular domain into the 60S subunit. While the globular domain almost exclusively interacts with conserved, interconnected rRNA segments of domains I and II of the 25S rRNA, the eukaryote-specific extension spans across more than half the width of the solvent-side 60S surface and thereby engages in an intricate network of interactions, primarily with eukaryote-specific rRNA and r-protein moieties (Fig 1A and 1B) [11,15,17]. The first part of the eukaryote-specific extension is accommodated by Rpl18 (eL18) and ES15^L^ (H45), and the second part is sandwiched between Rpl7 (uL30), mostly by its long, eukaryote-specific N-terminal α-helix, and helices ES7^L^c/ES7^L^b of ES7^L^ (Fig 1B). Moreover, the eukaryote-specific r-proteins Rpl20 (eL20) and Rpl21 (eL21) contact the C-terminal residues of Rpl4.

In this study, we show that both the long internal loop and the C-terminal eukaryote-specific extension are essential features of Rpl4. We further reveal that Rpl4 contains at least five distinct nuclear localization signals (NLS), which map to different regions of Rpl4, including the long internal loop and the C-terminal extension. Notably, we have identified a previously uncharacterized protein, termed Acl4, as a specific binding partner of Rpl4. Acl4 interacts with the C-terminal part of the long internal loop of Rpl4 and both genetic and biochemical evidence suggests that Acl4 can be considered as a dedicated chaperone of Rpl4. Furthermore, we show that Acl4, which localizes to the cytoplasm and the nucleus, has the capacity to capture nascent Rpl4 in a co-translational manner. Taken together, our data indicate that the dedicated chaperone Acl4 accompanies Rpl4 from the cytoplasm to its pre-60S assembly site in the nucleus.

## Results

### The long internal loop and the C-terminal extension of Rpl4 fulfil essential functions

In *S*. *cerevisiae*, the paralogous *RPL4A* and *RPL4B* genes encode the essential r-protein Rpl4 of 362 amino acids length whose two versions, Rpl4a and Rpl4b, only differ at amino acid position 356 (threonine *versus* alanine). Notably, Rpl4 is composed of a globular domain, which contains a small (amino acids 184–205) and a long internal loop (amino acids 44–113), and a eukaryote-specific C-terminal extension (amino acids 264–362) (see Introduction and Fig 1). To determine the contribution of the long internal loop and the C-terminal extension to Rpl4 function, we selected Rpl4a since *Δrpl4a* null mutant cells, but not *Δrpl4b* null mutant cells, showed a moderate growth defect and reduced steady-state levels of 60S subunits, as evidenced by a shortage of free 60S subunits and the accumulation of half-mer polysomes (S2 Fig). For phenotypic analysis, plasmids encoding the Rpl4a deletion variants were transformed into a *Δrpl4a*/*Δrpl4b* strain harbouring an instable *URA3*/*ADE3* plasmids containing *RPL4A* (*RPL4* shuffle strain). Importantly, expression of wild-type Rpl4a under the control of its cognate promoter from a monocopy plasmid in the *RPL4* shuffle strain conferred, upon plasmid shuffling on 5-FOA containing plates, almost wild-type growth and resulted only in a very minor 60S deficiency (S2 Fig). Complete deletion of the C-terminal extension (N264 construct; *i*.*e*.: Rpl4a deletion variant consisting of amino acids 1–264) did not support growth (Fig 1C). To map more precisely the important regions within the C-terminal extension, we generated a series of progressive C-terminal deletion variants. This analysis revealed that the last 30 amino acids (N332 construct) of Rpl4 are completely dispensable; conversely, removal of the C-terminal 71 amino acids (N291 construct) resulted in lethality (Fig 1C). The first *rpl4* truncation mutant showing a slow-growth phenotype lacked the last 37 amino acids (N325 construct) and further deletion led to a severe slow-growth phenotype (N312 and N301 constructs) (Fig 1C and S3 Fig). Moreover, the severity of the observed growth defects correlated with the extent of the deficiency in 60S subunits, as indicated by the more dramatic reduction in polysome content in Rpl4.N312 and Rpl4.N301 expressing cells (S3 Fig). All the viable C-terminal deletion variants localized, as also observed for N-terminally yEGFP-tagged Rpl4a, mainly to the cytoplasm (S3 Fig). While removal of the small internal loop (deletion of amino acids 185–200) did not affect growth, deletion of the long internal loop (deletion of amino acids 46–110) resulted in a non-functional Rpl4 protein (Fig 1C). We conclude that, in agreement with its central location within 60S subunits, the presence of the long internal loop is strictly required for the synthesis of functional 60S subunits. Concerning the role of the eukaryote-specific C-terminal extension, we conclude that its interaction with Rpl20 and Rpl21 is dispensable for full functionality of Rpl4. More importantly, the interaction network formed by the second part of the C-terminal extension (from amino acids 308 onwards) with Rpl7 and ES7^L^b/c contributes majorly, as evidenced by the severe slow-growth and the temperature sensitivity of the Rpl4a.N301 and Rpl4a.N312 constructs (Fig 1C and S3 Fig), to the efficient recruitment of Rpl4 and/or the assembly of functional 60S subunits. Further deletion of Rpl4 sites (amino acids 292–301) that mediate some of the interactions with Rpl18 and ES15^L^ conferred lethality; thus, indicating an additional important role of these contacts for incorporation of Rpl4, pre-60S assembly and/or the functional integrity of 60S subunits (see also Discussion). At this point however, it cannot be ruled out that the lethality of Rpl4a.N291 and Rpl4a.N264 variants may not be simply due to their inefficient nuclear import.

### Rpl4 contains several nuclear localization signals

**Fig 2:**
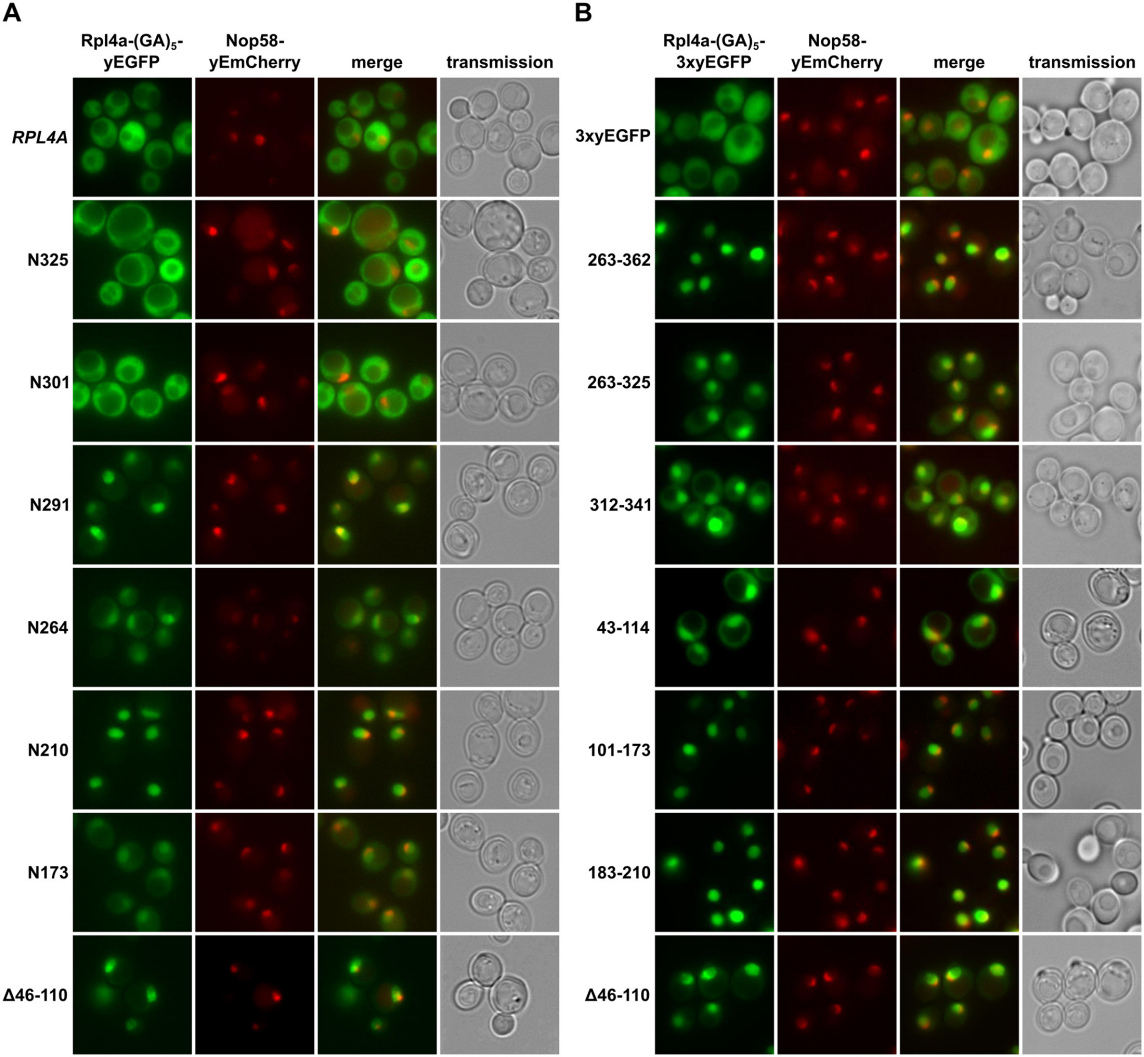
Rpl4 harbours five distinct nuclear localization signals. **A,** Subcellular localization of C-terminally truncated Rpl4a proteins. Plasmids expressing wild-type Rpl4a-(GA)_5_-yEGFP or the indicated *rpl4a* truncation variants from the cognate *RPL4A* promoter were transformed into a wild-type strain expressing the nucleolar marker protein Nop58-yEmCherry from the genomic locus. Transformed cells were grown in SC-Leu medium at 30°C and inspected by fluorescence microscopy. **B,** Mapping of nuclear localization signals within Rpl4a. Plasmids expressing, under the transcriptional control of the *ADH1* promoter, the (GA)_5_-3xyEGFP control protein or the indicated Rpl4a fragments fused, *via* a (GA)_5_-linker, to a C-terminal 3xyEGFP were transformed into a wild-type strain expressing the nucleolar marker protein Nop58-yEmCherry from the genomic locus. Transformed cells were grown in SC-Leu medium at 30°C and inspected by fluorescence microscopy.

To determine whether the lethal C-terminal deletion variants of Rpl4 could still enter the nucleus, we expressed them from plasmid, under the transcriptional control of the cognate promoter, as fusion proteins with a C-terminal yEGFP in a wild-type strain containing the nucleolar marker protein Nop58-yEmCherry. While Rpl4.N291 localized almost exclusively to the nucleus, Rpl4.N264 showed clear nuclear enrichment but also some cytoplasmic signal (Fig 2A). Further C-terminal deletion revealed that constructs Rpl4.N210 and Rpl4.N173 exhibited a complete or partial nuclear accumulation. On the other hand, and as observed above (S3 Fig), the viable C-terminal deletion constructs displayed a mainly cytoplasmic localization (Fig 2A), indicating that they are assembled into mature 60S subunits. Conversely, an Rpl4 construct lacking the long internal loop displayed a striking nuclear enrichment, suggesting that the presence of the long internal loop is required for the incorporation of Rpl4 into and/or the nuclear maturation of pre-60S subunits (see below and Discussion). Furthermore, we conclude that the Rpl4 variants lacking completely or more than two-thirds of the C-terminal extension (N264 and N291 constructs) enter the nucleus but are presumably not efficiently assembled into pre-60S subunits.

To obtain a complete overview of the different Rpl4 regions that may confer nuclear localization, we next wished to precisely map the individual NLSs. To reduce passive diffusion across the NPC, we fused a C-terminal triple yEGFP (3xyEGFP) preceded by a short (GA)_5_ linker, consisting of five glycine-alanine repeats, to the different Rpl4 fragments. Since an Rpl4 fragment consisting of the complete C-terminal extension (263C construct; amino acids 263–362) localized to the nucleus (Fig 2B), we first determined the NLS region(s) within the C-terminal extension. This analysis revealed that the C-terminal extension contains two distinct, but partially overlapping regions, consisting of amino acids 263–325 and 312–341, which conferred nuclear targeting, albeit slightly less efficiently than amino acids 263–362 (Fig 2B). These two regions can be considered as minimal NLSs since their further N- or C-terminal shortening resulted in a mostly cytoplasmic signal (S4 Fig). In agreement with the exclusive nuclear localization of the N210 construct (Fig 2A), we could map a strong NLS to amino acids 183–210, which basically encompasses the small internal loop region of Rpl4 (Fig 2B). Despite the finding that the N173 construct only displayed a partial nuclear enrichment, we could identify therein two quite efficient, but again partially overlapping, NLS regions consisting of amino acids 43–114 and 101–173 (Fig 2B). Since their further N- and/or C-terminal shortening strongly decreased the intensity of the nuclear signal (S4 Fig), these two regions likely correspond to minimal NLSs. Notably, the NLS region defined by amino acids 43–114 corresponds to the long internal loop of Rpl4. Taken together, our comprehensive analysis revealed that Rpl4 contains at least five distinct NLSs, with four of these being part of two larger, overlapping NLS regions (Fig 2A and S4 Fig). However, due to the enormous combinatorial complexity—there are ten importin-β proteins in yeast, which, moreover, often display significant overlap in substrate specificity [44]–we have not attempted to assign the importin(s) responsible for the nuclear import of the five individual NLSs within Rpl4.

### The C-terminal extension contributes to incorporation of Rpl4 into pre-60S subunits

**Fig 3:**
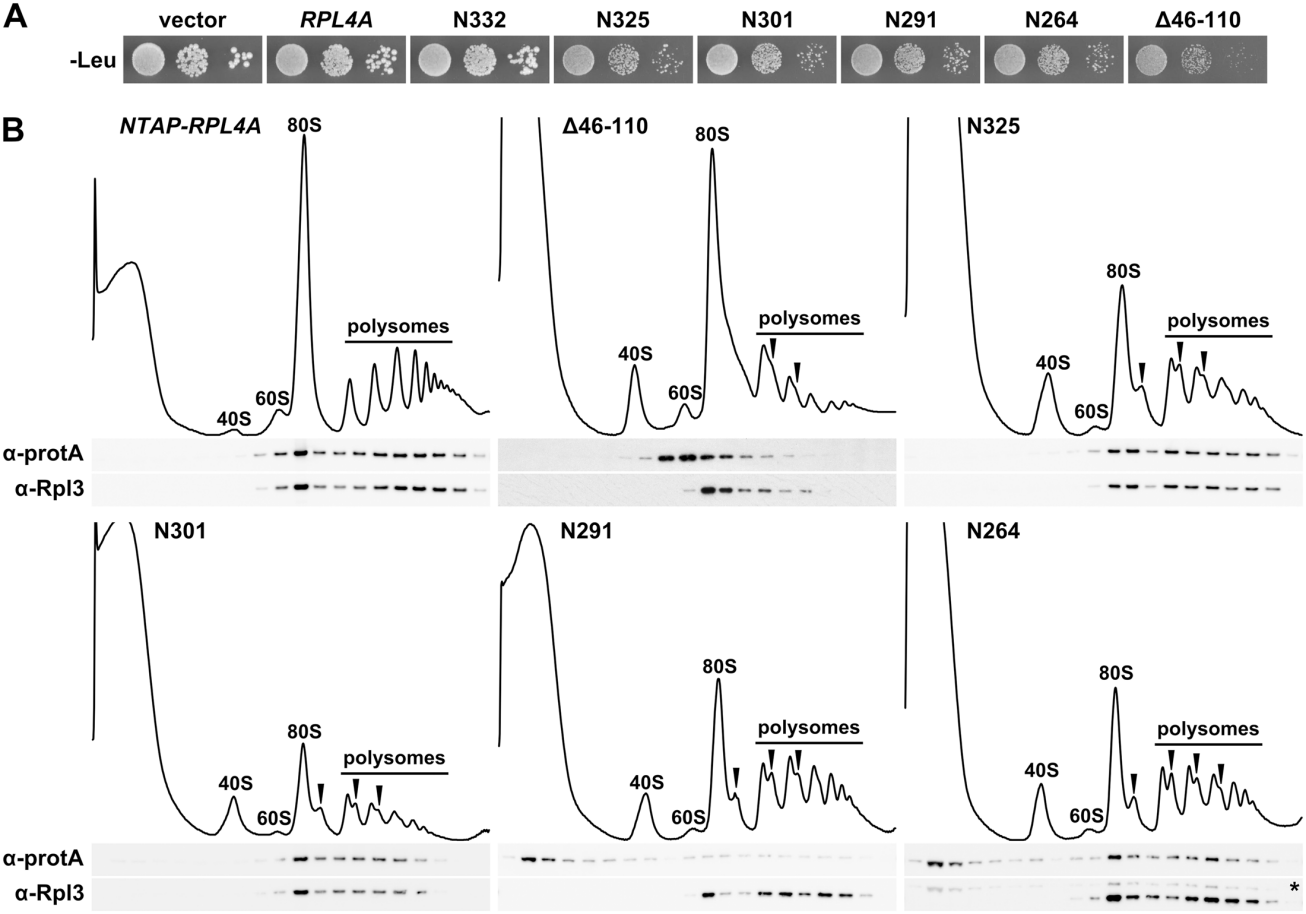
The C-terminal extension contributes to the efficient incorporation of Rpl4 into 60S subunits. **A,** Expression of C-terminally truncated Rpl4a variants confers a dominant slow-growth phenotype. Empty vector (YCplac111) and plasmid-borne wild-type *RPL4A* or the indicated *rpl4a* deletion mutants, expressed under the control of the cognate promoter, were transformed into the haploid wild-type strain YDK11-5A. Transformants were restreaked and cells were spotted in 10-fold serial dilution steps onto SC-Leu (-Leu) plates, which were incubated for 3 d at 30°C. **B,** Analysis of the incorporation of C-terminally truncated Rpl4 variants into 60S subunits and translating ribosomes. Whole cell lysates, derived from wild-type cells expressing N-terminally TAP tagged Rpl4a or the indicated Rpl4a deletion variants, were prepared under polysome-preserving conditions in the presence of cycloheximide and analyzed by sucrose gradient centrifugation and fractionation. Five A_260_ units were resolved in 10–50% sucrose gradients and the absorption profiles were recorded by continuous monitoring at A_254_ (upper panels). Sedimentation is from left to right. The peaks of free 40S and 60S subunits, 80S free couples/monosomes, and polysomes are indicated. Half-mers are highlighted by arrowheads. Gradient fractions were subjected to Western blot analysis using anti-protA and anti-Rpl3 antibodies (lower panels). The asterisk indicates the residual NTAP-Rpl4a.N264 signal in this anti-Rpl3 Western blot.

To explore a possible role of the C-terminal extension for assembly of Rpl4 into pre-60S particles, we first expressed the partially functional (N325 and N301 constructs) and lethal (N291 and N264 constructs) C-terminal deletion variants from plasmid, under the transcriptional control of the cognate promoter, in a wild-type strain and examined their effects on growth. While additional expression of Rpl4a did not affect the growth of wild-type cells, all of the above C-terminal deletion variants conferred a similar slow-growth phenotype and decrease in 60S and polysome content (Fig 3A and 3B). Moreover, related growth defects were observed when these C-terminal deletion variants were overexpressed from a galactose-inducible promoter (S5 Fig). In agreement with their cytoplasmic localization, the partially functional Rpl4.N325 and Rpl4.N301 proteins, expressed as fusion proteins with an N-terminal TAP tag in wild-type cells, got incorporated into 60S, 80S, and translating ribosomes (Fig 3B). Conversely, the almost exclusively nuclear Rpl4.N291 only showed a very minor 60S association and was mostly detectable in the soluble fractions (Fig 3B), while Rpl4.N264, as already suggested by its dual cytoplasmic and nuclear localization, was both present in the soluble and ribosome-associated fractions (Fig 3B). We conclude that the C-terminal extension contributes to the efficient assembly of Rpl4 into nuclear pre-60S particles. Interestingly, expression of Rpl4.N291, which is not stably incorporated into 60S subunits, confers a severe slow-growth phenotype to wild-type cells. This observation raised the possibility that free Rpl4.N291 may titrate a protein that acts positively on wild-type Rpl4. Finally, the Rpl4 protein lacking the long internal loop mainly migrated in the fractions surrounding the 60S peak and exhibited a clearly reduced 80S and polysome distribution when compared to Rpl3 (Fig 3B). Considering that the Rpl4(Δ46–110) protein localized predominantly to the nucleus (Fig 2A), we conclude that this Rpl4 variant gets efficiently incorporated into pre-60S subunits and, therefore, that the long internal loop is not a critical determinant for the assembly of Rpl4. Moreover, expression of Rpl4(Δ46–110) in wild-type cells not only conferred a slow-growth phenotype but also some decrease in 60S subunits and a drastic reduction in polysome content (Fig 3A and 3B); thus, pointing to a possible role of the long internal loop in maturation events that are necessary for the productive assembly of export-competent pre-60S subunits (see Discussion).

### Identification of Acl4 as a specific binding partner of free Rpl4

**Fig 4:**
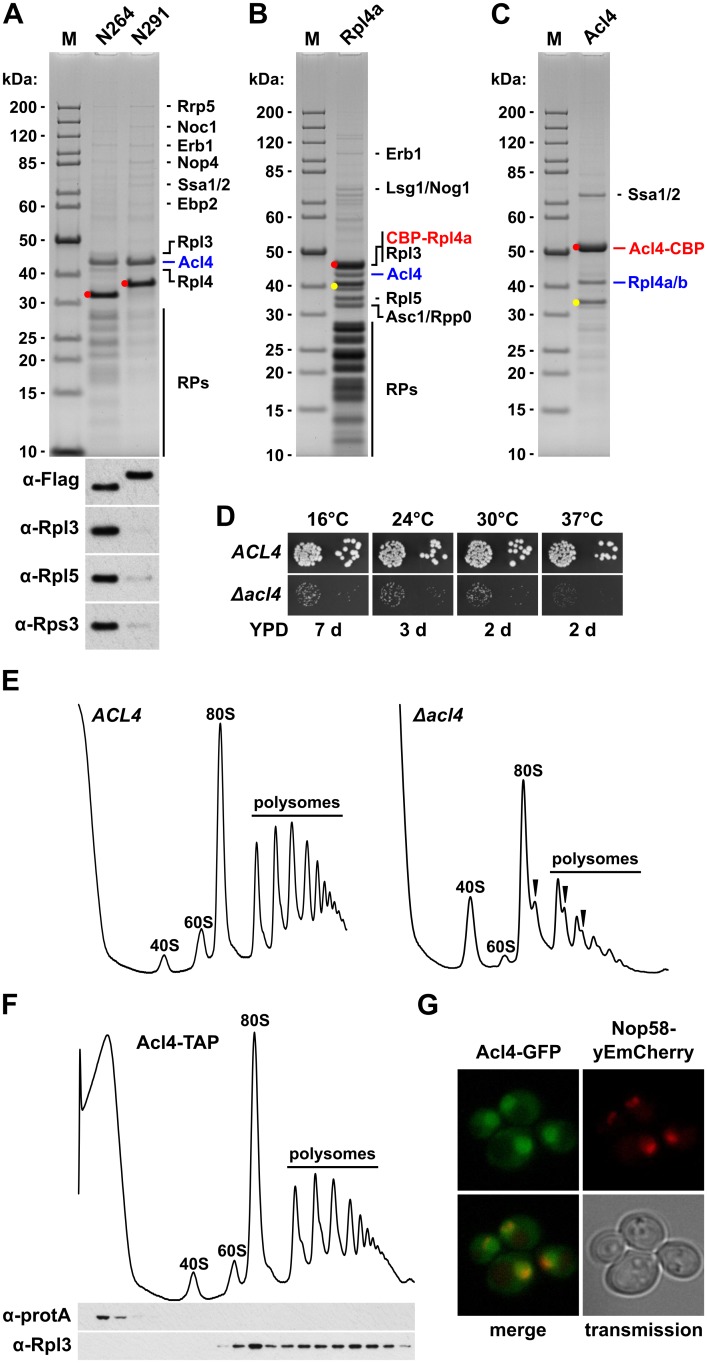
Rpl4 is associated with the specific binding partner Acl4. **A,** Identification of Acl4 as a binding partner of Rpl4. Tandem-affinity purification of N-terminally TAP-tagged Rpl4a.N264 and Rpl4a.N291, expressed from a monocopy plasmid under the transcriptional control of the cognate promoter in a wild-type strain, from whole cell lysates. Final EGTA eluates were analyzed by SDS-PAGE and Coomassie staining (upper panel) and by Western blotting using anti-Flag (for revealing the bait proteins), anti-Rpl3, anti-Rpl5, and anti-Rps3 antibodies (lower panel). The position of the bait proteins is highlighted by a red dot. Proteins identified by mass spectrometry are indicated. The bands migrating in the low-molecular-weight range correspond to r-proteins (RPs). M, molecular weight standard. **B,** Rpl4a co-purifies 80S ribosomes and Acl4. Tandem-affinity purification of N-terminally TAP-tagged Rpl4a, expressed from a monocopy plasmid under the transcriptional control of the cognate promoter as the sole Rpl4 source in the *RPL4* shuffle strain YBP15, from whole cell lysates. Final EGTA eluates were analyzed by SDS-PAGE and Coomassie staining. The position of the bait protein and its most prominent degradation product is highlighted by a red and yellow dot, respectively. Proteins identified by mass spectrometry are indicated. **C,** Acl4 specifically co-purifies non-ribosome associated Rpl4. Tandem-affinity purification of C-terminally TAP-tagged Acl4, expressed from the genomic locus under the transcriptional control of the cognate promoter, from whole cell lysates. The position of the bait protein is highlighted by a red dot. Proteins identified by mass spectrometry are indicated. The band marked by a yellow dot contains C-terminally truncated Rpl4. **D,** The *Δacl4* null mutant displays a severe slow-growth phenotype. Cells of a wild-type (*ACL4*) and an isogenic *Δacl4* null mutant *(Δacl4*) strain were spotted in 10-fold serial dilution steps onto YPD plates, which were incubated for the indicated times at 16°C, 24°C, 30°C, and 37°C. **E,** Acl4 is required for the efficient production of 60S subunits. Polysome profiles of wild-type (*ACL4*) and isogenic *Δacl4* null mutant *(Δacl4*) cells grown at 30°C in YPD medium. Eight A_260_ units were resolved in 10–50% sucrose gradients and the absorption profiles were recorded by continuous monitoring at A_254_. Sedimentation is from left to right. The peaks of free 40S and 60S subunits, 80S free couples/monosomes, and polysomes are indicated. Half-mers are highlighted by arrowheads. **F,** Acl4 is not associated with 60S subunits or translating ribosomes. Whole cell lysates of an *ACL4*-TAP strain, expressing C-terminally TAP tagged Acl4 from the genomic locus under the transcriptional control of the cognate promoter, were prepared under polysome-preserving conditions and analyzed by sucrose gradient centrifugation and fractionation. Five A_260_ units were resolved in 10–50% sucrose gradients and the absorption profiles were recorded by continuous monitoring at A_254_ (upper panel). Gradient fractions were subjected to Western blot analysis using anti-protA and anti-Rpl3 antibodies (lower panel). **G,** Acl4 localizes to the cytoplasm and nucleus. The localization of genomically expressed Acl4-GFP was assessed by fluorescence microscopy in cells grown at 30°C in YPD medium. The localization of the nucleolar marker protein Nop58-yEmCherry, also expressed from its genomic locus, serves to indicate the subcellular position of the nucleus.

In order to corroborate the sucrose gradient fractionation data and, if possible, to identify the anticipated binding partner of free Rpl4, we performed tandem-affinity purifications (TAP) of N-terminally TAP-tagged Rpl4.N264 and Rpl4.N291. In agreement with its dual gradient localization, purification of NTAP-Rpl4.N264 not only yielded the bait protein but also sub-stoichiometric amounts of r-proteins (Fig 4A). Most strikingly, a prominent band, corresponding to a protein migrating at around 43 kDa, was observed in the final EGTA eluates of both purifications (Fig 4A). Mass spectrometric analysis revealed that this band contained the previously uncharacterized protein Ydr161w, which was recently assigned the name Acl4 (Assembly Chaperone of RpL4) [45]. Moreover, both purifications contained low levels of early 60S biogenesis factors (Fig 4A), indicating that both Rpl4.N264 and Rpl4.N291 have the capacity to be incorporated into nucleolar pre-60S particles. In further validation of an association with early pre-60S particles, N-terminally GFP-tagged Rpl4a.N264 precipitated the 27SA_2_ and 27SB pre-rRNAs (S6 Fig). To address whether Acl4 was also associated with full-length Rpl4 under normal conditions, we expressed N-terminally TAP-tagged full-length Rpl4a from plasmid, under the transcriptional control of the cognate promoter, in *Δrpl4a*/*Δrpl4b* cells. In agreement with the good functionality of this construct (S7 Fig), the purification revealed that NTAP-Rpl4a was efficiently incorporated into mature 60S subunits (Fig 4B). Notably, significant amounts of Acl4 could be co-purified with the NTAP-Rpl4a bait. Next, we purified in a reciprocal experiment C-terminally TAP-tagged Acl4, which was expressed as a fully functional protein from its genomic locus (S7 Fig), thereby revealing that Acl4 specifically co-purified free Rpl4 (Fig 4C). Moreover, the only other prominent band, migrating slightly above 70 kDa, contained the Hsp70 chaperones Ssa1, Ssa2, Ssa3, and Ssa4. However, we have not further explored the functional significance of their co-enrichment with the Acl4-TAP bait in this study, since Hsp70 chaperones, belonging to the Ssa and Ssb subfamilies, are rather commonly found in TAP purifications. Altogether, these *in vivo* purifications provide strong evidence that Acl4 is a specific binding partner of non-ribosome bound Rpl4.

Acl4 is an acidic protein (pI 4.15) of 387 amino acids with a calculated molecular mass of 42.94 kDa. The eukaryote-specific Acl4 is predicted to contain 16 α-helices that, based on our bioinformatics analysis, may form up to eight tetratrico peptide repeats (TPR) or TPR-like repeats. Notably, TPR domains build the scaffolds that mediate protein-protein interactions and the assembly of multiprotein complexes in a versatile manner [46,47]. Acl4 is not only conserved in fungi, such as filamentous ascomycetes (*e*.*g*.: *Chaetomium thermophilum*, Accession: XP_006691536) and fission yeasts (*e*.*g*.: *Schizosaccharomyces pombe*, Accession: NP_596496), as orthologues can also be found in protists (*e*.*g*.: *Trypanosoma brucei*, Accession: XP_011776831), arachnids (*e*.*g*.: *Stegodyphus mimosarum*, Accession: KFM57536 and *Metaseiulus occidentalis*, Accession: XP_003742882), ray-finned and coelcanth fish (*e*.*g*.: *Danio rerio*, Accession: XP_009290679 and *Latimeria chalumnae*, Accession: XP_006002768), and amphibians (*e*.*g*.: *Xenopus tropicalis*, Accession: XP_002934631). However, there are no orthologues in the evolutionary more advanced classes of reptiles, birds, and mammals. Moreover, Acl4 is not present in plants (*e*.*g*.: *Arabidopsis thaliana*), insects (*e*.*g*.: *Drosophila melanogaster*), and nematodes (*e*.*g*.: *Caenorhabditis elegans*), indicating that Acl4 can also be specifically lost in earlier evolutionary branches.

To address the functional significance of the identification of Acl4 as an Rpl4 binding protein, we first determined whether Acl4 contributed to the biogenesis of 60S subunits. Haploid cells with a chromosomally disrupted *ACL4* gene *(Δacl4*) were viable, but displayed a severe slow-growth phenotype at all tested temperatures (Fig 4D). In agreement with an involvement in 60S biogenesis, polysome profile analysis revealed that *Δacl4* cells contained reduced levels of 60S subunits, as evidenced by a shortage of free 60S subunits and the accumulation of half-mer polysomes, resulting in a substantial decrease in overall polysome content (Fig 4E). Compared to cells that were genetically depleted for Rpl4, which showed as previously reported a striking decrease in 27SB and 7S pre-rRNAs (S8 Fig) [43,48], the reduction of these pre-rRNA species was clearly observable but less pronounced in *Δacl4* cells (S8 Fig). In line with the *in vivo* purification, sucrose gradient fractionation revealed that Acl4-TAP was exclusively present in the soluble fractions (Fig 4F); thus, providing further evidence that Acl4 is not stably associated with pre-60S or mature 60S subunits. Finally, Acl4-GFP, expressed from its genomic locus as a functional protein at 30°C (S7 Fig), localized to both the nucleus and cytoplasm (Fig 4G). Since Acl4 lacks a predicted NLS, it is highly likely that Acl4 may already bind to Rpl4 in the cytoplasm and may be imported in complex with Rpl4, which contains several experimentally established NLS regions (Fig 2B).

### Acl4 interacts with the long internal loop of Rpl4

**Fig 5:**
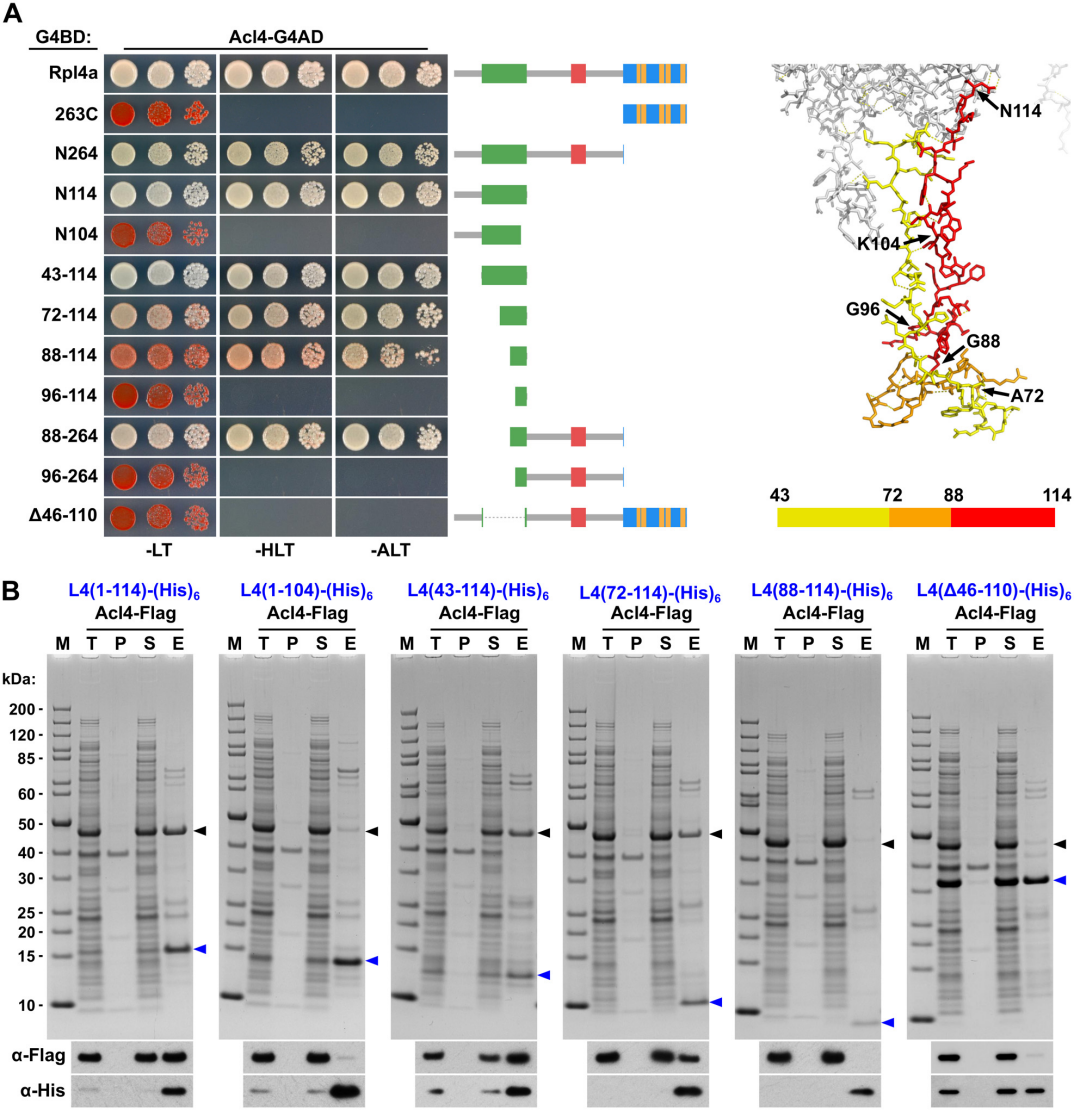
Acl4 interacts with the C-terminal part of the long internal loop of Rpl4. **A,** Mapping of the Acl4 binding site on Rpl4a by the yeast two-hybrid (Y2H) interaction assay (left panel). Plasmids expressing full-length Rpl4a or the indicated Rpl4a fragments, fused to the C-terminal Gal4 DNA-binding domain (G4BD), and full-length Acl4, fused to the C-terminal Gal4 activation domain (G4AD), were co-transformed into the Y2H reporter strain PJ69-4A. Cells were spotted in 10-fold serial dilution steps onto SC-Leu-Trp (-LT), SC-His-Leu-Trp (-HLT), and SC-Ade-Leu-Trp (-ALT) plates, which were incubated for 3 d at 30°C. The different Rpl4a constructs are schematically depicted on the right; the colour code to indicate the different features of Rpl4 is as in Fig 1C. Close-up view of the long internal loop of Rpl4 (right panel). The minimal Acl4 binding site on Rpl4a, as determined by Y2H and *in vitro* binding assays, is coloured in red (amino acids 88–114; Y2H) and orange/red (amino acids 72–114; *in vitro*), respectively. **B,** *In vitro* binding assay between Rpl4a and Acl4. The indicated C-terminally (His)_6_-tagged Rpl4a variants and full-length Acl4-Flag were co-expressed in *E*. *coli* and purified *via* Ni-affinity purification. Proteins were revealed by SDS-PAGE and Coomassie staining (top) or by Western blot analysis using anti-Flag (Acl4-Flag) and anti-His (Rpl4a-(His)_6_ variants) antibodies (bottom). T, total extract; P, pellet fraction (insoluble proteins); S, soluble extract; E, imidazole eluate; M, molecular weight standard. Blue arrowheads highlight the bands corresponding to the different Rpl4a-(His)_6_ variants used as baits for the purifications. Black arrowheads indicate the position of Acl4-Flag.

To precisely map the binding site of Acl4 on Rpl4, we first conducted yeast two-hybrid (Y2H) interaction assays (Fig 5A). As already indicated by the co-purification of Acl4 with NTAP-Rpl4.N264 (Fig 4A), the C-terminal extension was neither required for nor sufficient to mediate the interaction with Acl4 (Fig 5A). Further C-terminal deletion analysis revealed that the first 114 amino acids (N114 construct) were sufficient for a robust interaction, while the Rpl4.N104 construct did not show any interaction with Acl4 (Fig 5A). We therefore tested next whether Acl4 recognized the long internal loop and, indeed, a strong Y2H interaction was observed between Acl4 and amino acids 43–114 of Rpl4. By shortening the long internal loop from the N-terminal side, amino acids 88–114 were identified as the minimal, albeit less efficient, interaction fragment. However, amino acids 88–264 resulted in an interaction that was almost as strong as the one between Acl4 and full-length Rpl4, thus indicating that the region around amino acid 88 likely corresponds to the N-terminal border of the minimal interaction fragment. In further support of this notion, amino acids 96–114 or 96–264 of Rpl4 were insufficient to promote an interaction with Acl4. Finally, deletion of the long internal loop from full-length Rpl4 (deletion of amino acids 46–110) completely abolished the Y2H interaction. To corroborate the Y2H data, we turned to *in vitro* binding assays (Fig 5B). To this end, we co-expressed C-terminally (His)_6_-tagged Rpl4 or fragments thereof with full-length Acl4-Flag in *Escherichia coli* and subsequently performed Ni-affinity purification of the different Rpl4 baits. These binding assays confirmed that the long internal loop contained the Acl4 binding site and defined amino acids 72–114 of Rpl4 as the minimal region conferring a robust interaction (Fig 5B). In contrast to the Y2H data, however, amino acids 88–114 of Rpl4 were insufficient to mediate the interaction *in vitro*. Finally, the Rpl4 bait lacking the long internal loop did not yield co-purification of Acl4 (Fig 5B).

To corroborate the *in vitro* binding data obtained with the *S*. *cerevisiae* proteins and to obtain, if possible, structural insight into the Acl4-Rpl4 interaction, we turned to the orthologous proteins from the thermophilic, filamentous ascomycete *C*. *thermophilum* (*ct*). Proteins from this organism often display excellent biochemical properties and are well suited for structural studies [49]. In agreement with being a functional orthologue, *ct*Acl4 complemented the slow-growth phenotype of *Δacl4* mutant cells to the wild-type extent (S9 Fig). Ni-affinity purification of *ct*Rpl4-(His)_6_ yielded stoichiometric amounts of co-expressed *ct*Acl4-Flag (S9 Fig). Moreover, these *in vitro* binding assays also confirmed that the long internal loop is sufficient to mediate the interaction. Most notably and in contrast to the *in vitro* binding data of the *S*. *cerevisiae* proteins, amino acids 89–115 of *ct*Rpl4 resulted in an efficient co-purification of *ct*Acl4-Flag (S9 Fig), suggesting that these residues indeed constitute the minimal, evolutionary conserved binding site for Acl4. Unfortunately however, we could so far not gain structural insights into the interaction mode, since our attempts to obtain crystals of full-length *ct*Acl4 or co-crystals of the *ct*Rpl4(89–115)/*ct*Acl4 complex were not yet successful.

**Fig 6:**
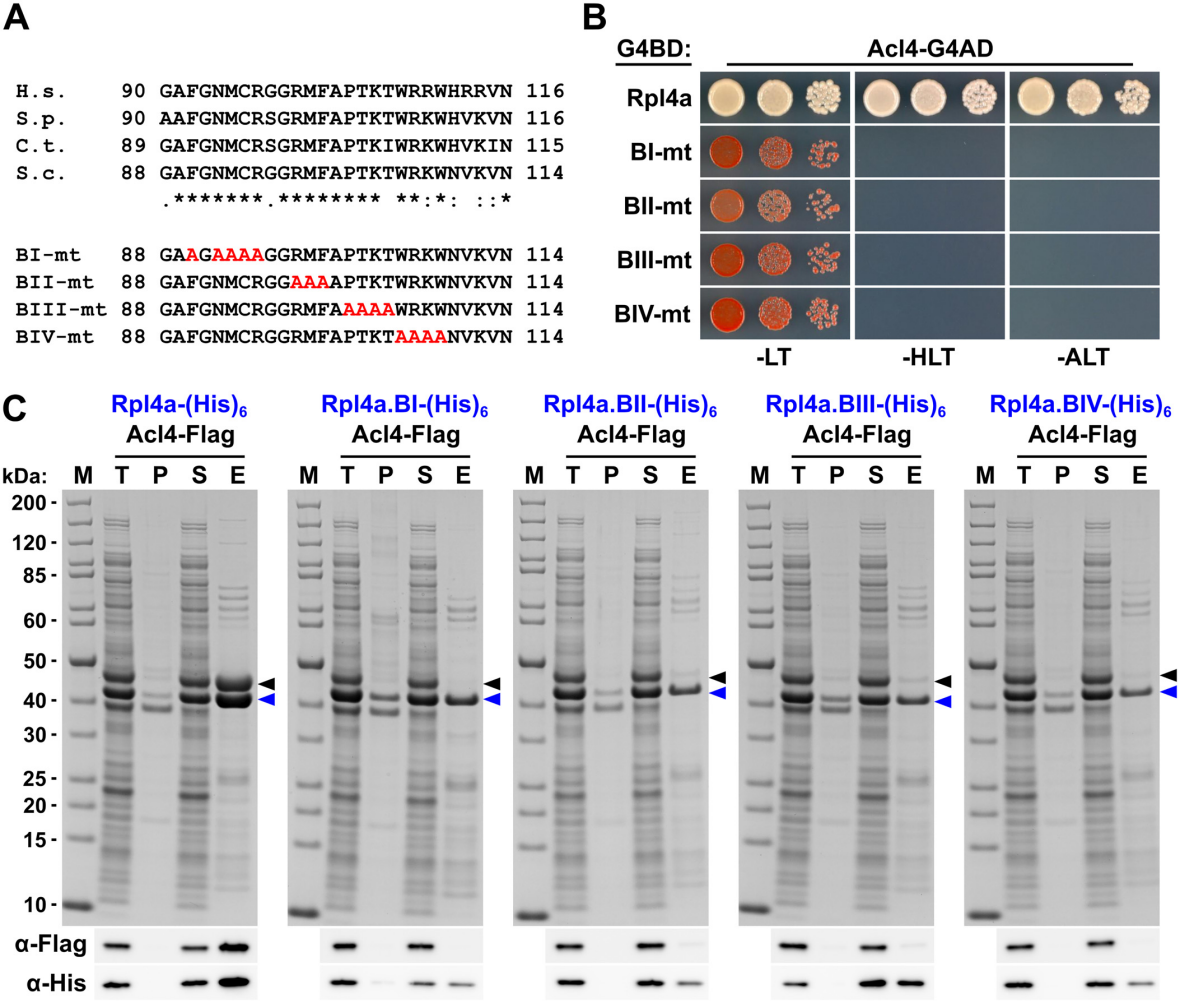
Multiple Rpl4 residues are required for the interaction with Acl4. **A,** Amino acid sequences of the minimal Acl4 binding site within the C-terminal part of the long internal loop of Rpl4 from different eukaryotic species (H.s., *Homo sapiens*; S.p., *Schizosaccharomyces pombe*; C.t., *C*. *thermophilum*; S.c., *S*. *cerevisiae*). Conserved (*), strongly similar (:), and weakly similar (.) amino acids are indicated below the alignment. The non-overlapping, consecutive alanine substitutions within this Rpl4a segment are depicted in the lower part: block-I mutant (BI-mt): F90A/N92A/M93A/C94A/R95A, block-II mutant (BII-mt): R98A/M99A/F100A, block-III mutant (BIII-mt: P102A/T103A/K104A/T105A, and block-IV mutant (BIV-mt): W106A/R107A/K108A/W109A). **B,** Y2H interaction between the Rpl4 alanine block mutants and Acl4. Plasmids expressing full-length Rpl4a or the indicated Rpl4a mutant variants, fused to the C-terminal Gal4 DNA-binding domain (G4BD), and full-length Acl4, fused to the C-terminal Gal4 activation domain (G4AD), were co-transformed into the Y2H reporter strain PJ69-4A. Cells were spotted in 10-fold serial dilution steps onto SC-Leu-Trp (-LT), SC-His-Leu-Trp (-HLT), and SC-Ade-Leu-Trp (-ALT) plates, which were incubated for 3 d at 30°C. **C,** *In vitro* interaction between the Rpl4 alanine block mutants and Acl4. Full-length Rpl4a or the indicated Rpl4a variants, containing a C-terminal (His)_6_ tag, were co-expressed with full-length Acl4-Flag in *E*. *coli* and purified *via* Ni-affinity purification. Proteins were revealed by SDS-PAGE and Coomassie staining (top) or by Western blot analysis using anti-Flag (Acl4-Flag) and anti-His (Rpl4a-(His)_6_ variants) antibodies (bottom). T, total extract; P, pellet fraction (insoluble proteins); S, soluble extract; E, imidazole eluate; M, molecular weight standard. Blue arrowheads highlight the bands corresponding to the different Rpl4a-(His)_6_ variants used as baits for the purifications. Black arrowheads indicate the position of Acl4-Flag.

To confirm that the C-terminal part of the long internal loop (amino acids 88–114) is required for the interaction in *S*. *cerevisiae* and to delineate important residues, we next generated four different Rpl4 variants harbouring non-overlapping, consecutive alanine substitutions (block-I: F90A/N92A/M93A/C94A/R95A, block-II: R98A/M99A/F100A, block-III: P102A/T103A/K104A/T105A, and block-IV: W106A/R107A/K108A/W109A) (see Fig 6A). Contrary to the complete deletion of the long internal loop (Fig 1C), all four alanine-block substitution mutants were viable, albeit displaying different degrees of growth deficiencies (S10 Fig). Moreover, these mutant Rpl4 proteins were similarly expressed as wild-type Rpl4 and their expression did not confer a slow-growth phenotype to wild-type cells (S10 Fig). Subsequent Y2H analyses and *in vitro* binding assays revealed that none of these four Rpl4 variants retained the capacity to interact with Acl4 (Fig 6B and 6C). Taken together, we conclude that Acl4 recognizes Rpl4 by directly interacting with the C-terminal part of the long internal loop (amino acids 88–114) and that, notably, residues dispersed along the entire, linear interaction segment are critical binding determinants.

### Acl4 acts as a chaperone of Rpl4

**Fig 7:**
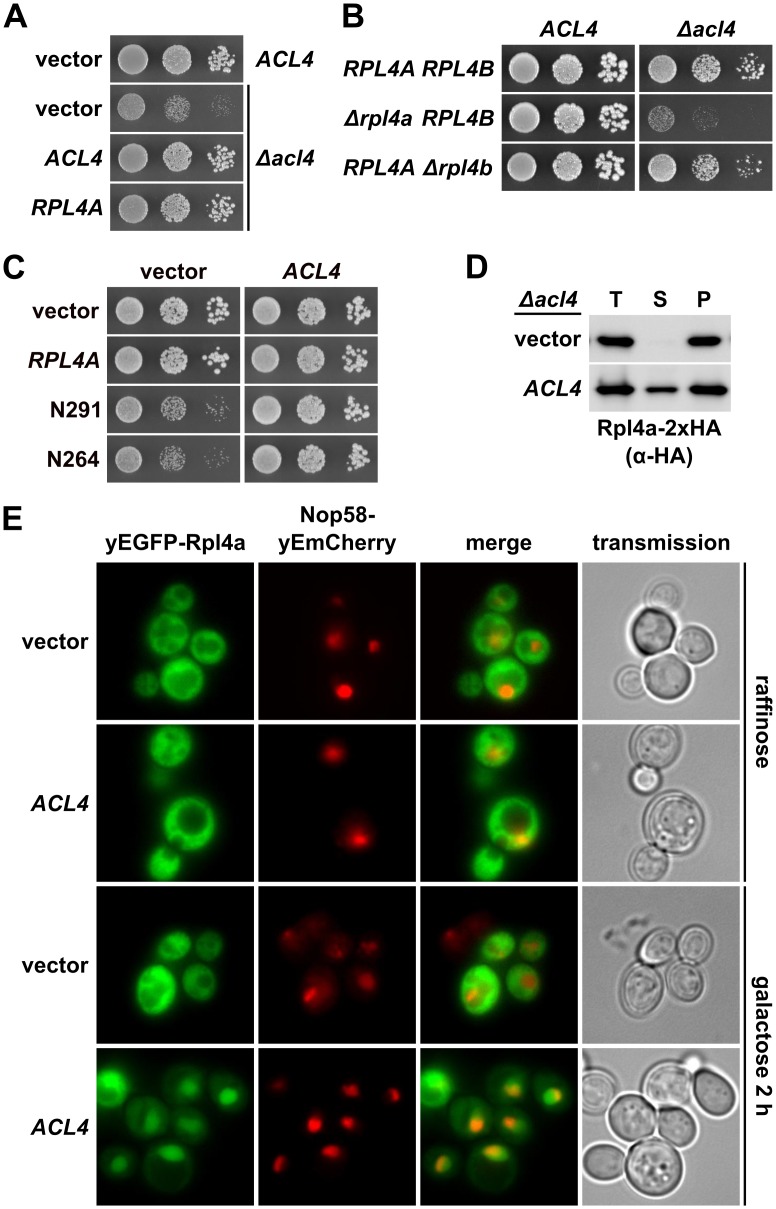
Acl4 ensures the sufficient availability of assembly-competent Rpl4. **A,** Increased dosage of Rpl4a suppresses the growth defect of *Δacl4* null mutant cells. The *Δacl4* null mutant was transformed with YCplac111 (empty vector), YCplac111-*ACL4*, and YCplac111-*RPL4A*. As a control, an isogenic wild-type strain was transformed with YCplac111. Transformants were restreaked and cells were spotted in 10-fold serial dilution steps onto SC-Leu plates, which were incubated for 2 d at 30°C. **B,** Absence of Rpl4a enhances the growth defect of *Δacl4* null mutant cells. Isogenic wild-type, *Δrpl4a*, and *Δrpl4b* cells either containing (*ACL4*) or lacking *(Δacl4*) the *ACL4* gene were spotted in 10-fold serial dilution steps onto a YPD plate, which was incubated for 2 d at 30°C. **C,** Overexpression of Acl4 suppresses the dominant-negative growth defect associated with expression of C-terminally truncated Rpl4a variants. The wild-type strain YDK11-5A was co-transformed with empty vector or plasmids expressing full-length Rpl4a or the indicated C-terminal deletion variants of Rpl4a, expressed from the cognate promoter, and empty vector or a multicopy plasmid expressing Acl4 from its authentic promoter. Transformants were restreaked and cells were spotted in 10-fold serial dilution steps onto SC-Leu-Trp plates, which were incubated for 2.5 d at 30°C. **D,** Acl4 is required for the soluble expression of Rpl4a in yeast. Expression of C-terminally 2xHA-tagged Rpl4a (Rpl4a-2xHA) was induced for 20 min from the *CUP1* promoter with 500 μM copper sulfate in *Δacl4* null mutant cells containing empty vector (vector) or a plasmid expressing Acl4 from the constitutive *ADH1* promoter (*ACL4*). After cell lysis with glass beads, extracts were centrifuged at 200’000 g for 1 h, and equal amounts of total extracts (T), soluble extracts (S), and pellet fractions (P) were analyzed by SDS-PAGE and Western blotting using an anti-HA antibody. **E,** Overexpression of Acl4 leads to nuclear accumulation of Rpl4a. A wild-type strain expressing Nop58-yEmCherry was co-transformed with a plasmid expressing N-terminally yEGFP-tagged Rpl4a from its cognate promoter and empty vector or a plasmid expressing Acl4 under the transcriptional control of the inducible *GAL1-10* promoter. Cells were grown in SC-Leu-Trp medium containing raffinose as carbon source and they were, prior to and after 2 h of galactose addition, inspected by fluorescence microscopy.

Having established Acl4 as a physical interaction partner of Rpl4, we next wished to assess the functional relevance of their association. Genetic analyses revealed that the slow-growth phenotype of *Δacl4* cells could be efficiently suppressed by overexpression of Rpl4a from a monocopy plasmid (Fig 7A). Moreover, cells simultaneously lacking Acl4 and Rpl4a exhibited a pronounced synthetic enhancement phenotype, as evidenced by their severely reduced growth rate compared to the one of *Δacl4* and *Δrpl4a* single mutant cells (Fig 7B). These findings point to an important function of Acl4 in ensuring that cells are provided with sufficient amounts of assembly-competent Rpl4. Along these lines, we observed that overexpression of Acl4 suppressed the growth defects associated with the expression of Rpl4.N264 and Rpl4.N291 (Fig 7C), which efficiently associate with Acl4 (Fig 4A) and therefore likely compete with endogenous Rpl4 for Acl4 binding. Finally, newly synthesized Rpl4-2xHA, expressed for 20 min from a copper-inducible promoter, was only found to be soluble in the presence, but not in the absence, of Acl4 (Fig 7D). Taken together, the genetic and biochemical evidence indicates that Acl4 can be considered as a specific chaperone of Rpl4.

### Acl4 is recruited to nascent Rpl4

**Fig 8:**
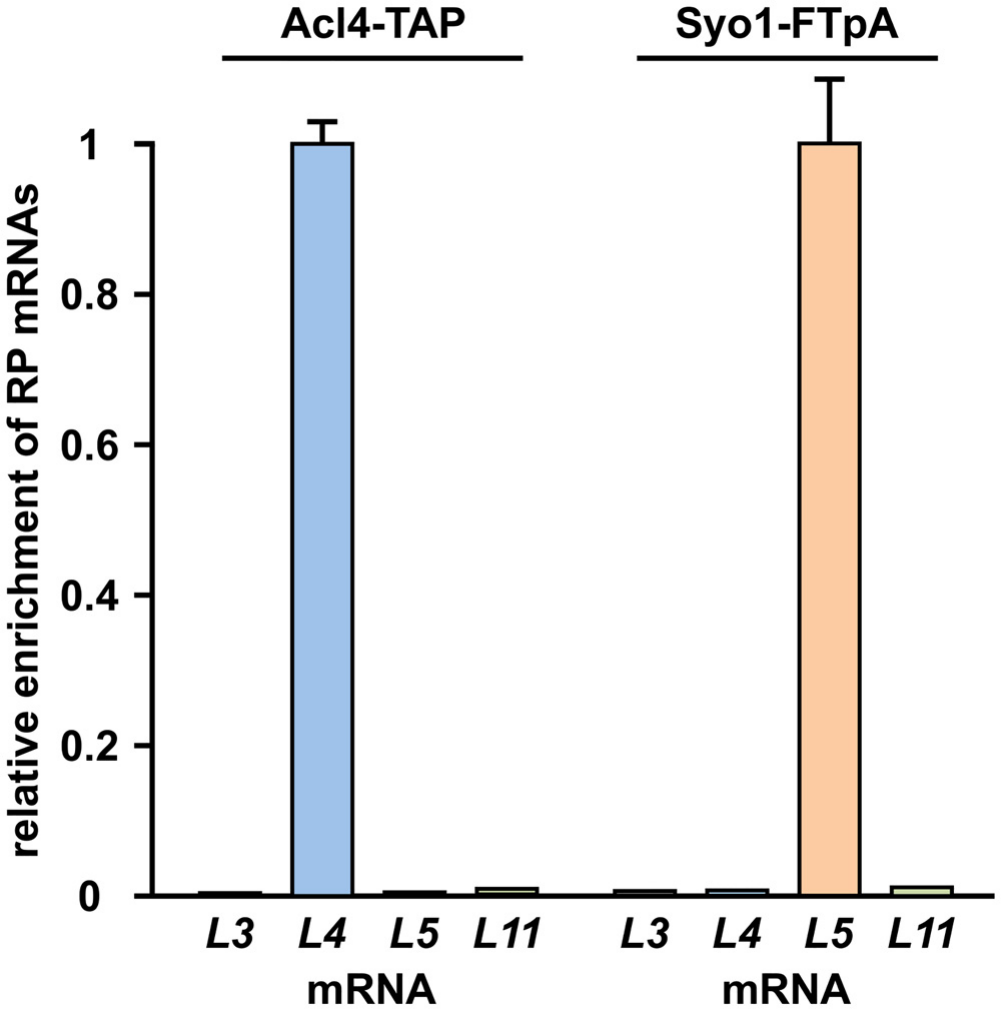
Co-translational capturing of Rpl4 by Acl4. The dedicated chaperones Acl4 (Acl4-TAP) and Syo1 (Syo1-FTpA) were affinity purified (IgG-Sepharose pull-down) from extracts of cycloheximide-treated cells and the associated RNA was isolated from the TEV eluates. Both chaperone purifications were assessed for their content of the *RPL3*, *RPL4*, *RPL5*, and *RPL11* r-protein mRNAs by real-time qRT-PCR. Note that real-time qRT-PCRs were performed in triplicate with all four oligonucleotide pairs using the same cDNA, derived from the total RNAs or the RNAs extracted from the TEV eluates. The data from one representative experiment are expressed as the relative enrichment of the specifically co-purified r-protein (RP) mRNA in each of the two chaperone purifications. Error bars showing the standard deviation indicate the quality of the technical replicate. A highly reproducible data set was obtained in an independent series of chaperone purifications.

Acl4, which lacks a predicted NLS, localizes both to the cytoplasm and nucleus (Fig 4G). Moreover, we observed that overexpression of Acl4 from a galactose-inducible promoter led to the nuclear accumulation of Rpl4 (Fig 7E). Therefore, Acl4 may already bind to Rpl4 in the cytoplasm and travel in complex with Rpl4 to the nucleus. To obtain evidence for a cytoplasmic interaction, which would most efficiently already be established during translation of Rpl4, we assessed whether Acl4 was recruited to nascent Rpl4. To this end, we employed, as recently described [38], a method coupling chaperone purification to the detection of associated mRNAs by real-time quantitative reverse transcription PCR (real-time qRT-PCR). Briefly, we purified Acl4-TAP and, as a control, Syo1-FTpA by IgG-Sepharose pull-down from extracts of cells that were, prior to harvesting, treated with cycloheximide in order to preserve the translating ribosomes on the mRNAs (see Methods). The purified chaperones were then released from the IgG-Sepharose beads by TEV cleavage and the associated RNA was extracted and transcribed into cDNA, which was used as the template for the assessment of the levels of the different r-protein mRNAs (*RPL3*, *RPL4*, *RPL5* and *RPL11*) by real-time qRT-PCR. As recently reported [38], purification of Syo1-FTpA specifically enriched the mRNA encoding Rpl5, while the amounts of the *RPL4* mRNA were similar to the one of the negative control mRNA *RPL3* (Fig 8). Interestingly, the mRNA encoding Rpl11, which forms together with Syo1 and Rpl5 a co-imported complex [34,35], was not enriched, indicating that only Rpl5, but not Rpl11, is captured by Syo1 in a co-translational manner. On the other hand, purification of Acl4-TAP yielded a specific and robust enrichment of the *RPL4* mRNA (Fig 8). Notably, the fold enrichment (around 150 fold) of the *RPL5* or *RPL4* mRNAs, compared to the non-specific r-protein mRNAs, was similar in the case of the Syo1-FTpA and Acl4-TAP purification, respectively. We conclude that Acl4 has the capacity to recognize Rpl4 in a co-translational manner.

## Discussion

**Fig 9:**
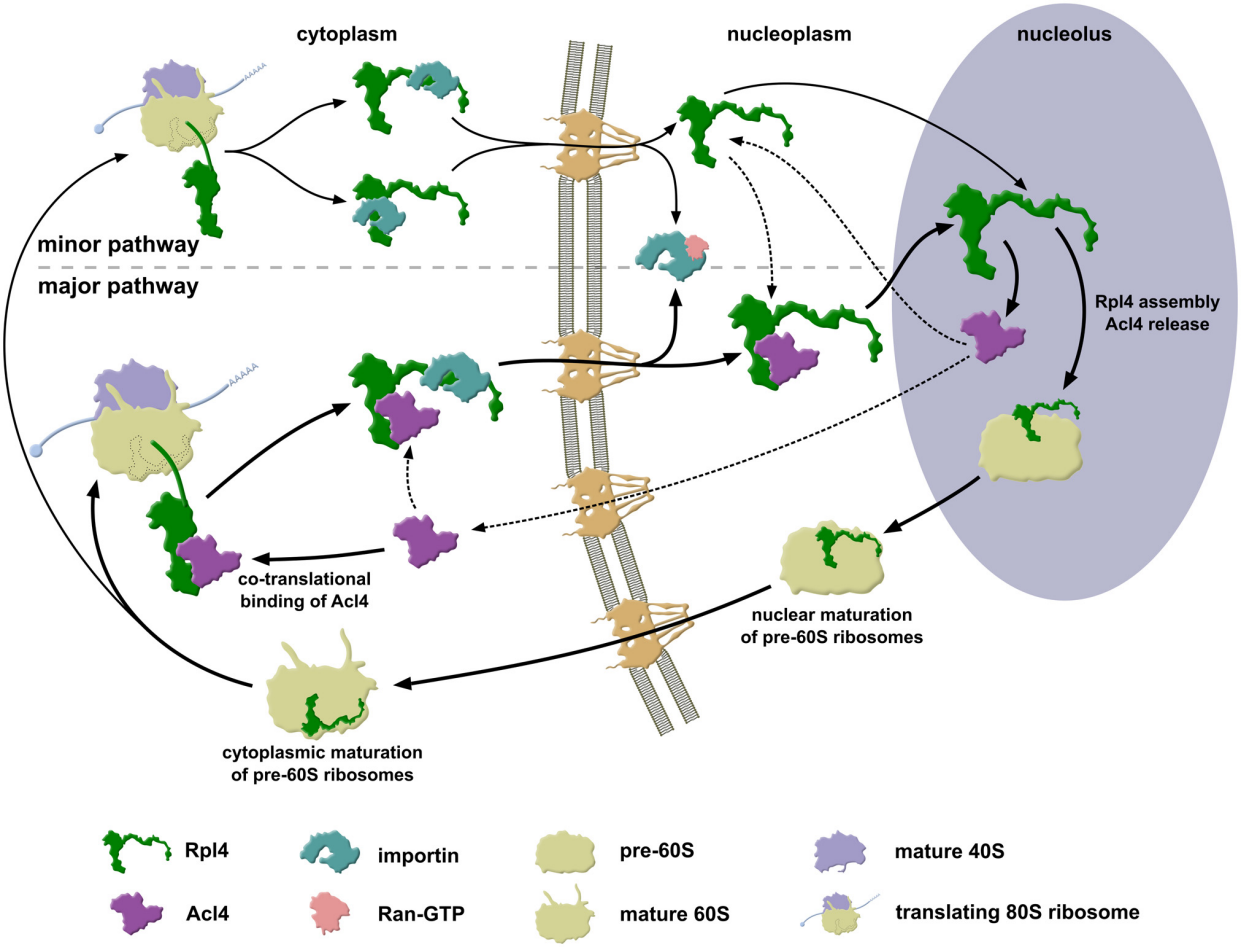
Model of Rpl4’s assembly path. Simplified model describing how the dedicated chaperone Acl4 accompanies Rpl4 to its nucleolar pre-60S assembly site. Acl4 associates with Rpl4 in the cytoplasm by recognizing, likely in a co-translational manner, the long internal loop of Rpl4. In this configuration, the eukaryote-specific extension of Rpl4, which harbours two overlapping nuclear localization signals (NLS), is available for binding to a nuclear transport receptor. Recent evidence indicates that the importin Kap104 may mediate the nuclear import of the Rpl4-Acl4 complex [45]; however, since Rpl4 contains additional NLSs and given the complexity of the NLS region within the eukaryote-specific extension, other importins may also have the capacity to transport free or Acl4-associated Rpl4. Upon appearance in the nucleus, Ran-GTP binds to the importin; thus liberating the Rpl4-Acl4 complex. Initial binding of Acl4-bound Rpl4 to early pre-60S particles likely occurs *via* Rpl4 surfaces that are not shielded by Acl4. Given that Acl4 is not detectably associated with pre-60S particles, the stable incorporation of the long internal loop, and hence the dissociation of Acl4 from Rpl4, are expected to proceed rapidly after the initial pre-60S docking of Rpl4. Once detached from Rpl4, Acl4 may either travel back to the cytoplasm, possibly by facilitated diffusion across the nuclear pore complex (NPC), to re-engage in another round of cytoplasmic recognition of Rpl4 and its fail-safe delivery to nucleolar pre-60S particles. Alternatively, Acl4 may remain in the nucleus and may be utilized to escort the Rpl4 fraction that was not imported in association with Acl4 to the pre-60S incorporation site. Correct assembly of Rpl4 permits the further nuclear maturation of pre-60S subunits, which are then exported to the cytoplasm where they engage, upon subunit joining, in translation. For more details, see Discussion.

Recent evidence has highlighted that r-proteins rely in several cases on specific binding partners, also referred to as dedicated chaperones, which favour the soluble expression of r-proteins, promote their nuclear import and/or coordinate their assembly into pre-ribosomal subunits (see Introduction). In the present study, we have identified such a specific binding partner, termed Acl4, which exclusively interacts with the LSU r-protein Rpl4. The genetic and biochemical data reported here indicate that Acl4 can be considered as a dedicated chaperone of Rpl4. Notably, Acl4 has the capacity to recognize, by directly interacting with the C-terminal part of the long internal loop, nascent Rpl4 in a co-translational manner. Further, the identification of several NLS regions within Rpl4 suggests that Acl4, which lacks a predicted NLS, gets imported into the nucleus in complex with Rpl4 and accompanies Rpl4 to its nucleolar pre-60S incorporation site. Finally, we show that both the eukaryote-specific C-terminal extension and the long internal loop are essential features of Rpl4, which are required for the assembly of Rpl4 into and the nuclear maturation of pre-60S subunits. While we compiled our manuscript, the Hurt laboratory, in collaboration with the Hoelz group, independently reported the identification and characterization of Acl4 as a binding partner and assembly chaperone of Rpl4 [45]. Notably, their study also described the partial crystal structure of *C*. *thermophilum* Acl4, revealing that, in agreement with our bioinformatics prediction, the central core of Acl4 is made up of 6.5 TPR repeats [45]. Moreover, the Woolford laboratory investigated in a recent study the contribution of the long internal loop and the eukaryote-specific C-terminal extension of Rpl4 to the assembly of 60S subunits [48]. By assimilating the data from these three different studies, we outline an integrated model, whose main steps are discussed below, describing how Acl4 may ensure the synthesis of assembly-competent Rpl4 and how the incorporation of Rpl4 into early pre-60S particles may proceed (Fig 9).

Several lines of evidence indicate that Acl4 can be considered as a dedicated ‘holding’ chaperone of Rpl4. First, Acl4 specifically binds to free Rpl4 and is therefore only, if at all, very transiently associated with pre-60S particles during the initial docking phase of Rpl4 (Fig 4C and 4F). Second, genetic experiments reveal an important function of Acl4 in ensuring that cells are provided with sufficient amounts of assembly-competent Rpl4 (Fig 7A and 7B) [45]. Suppression of the growth defects, entailed by the absence of a dedicated chaperone, by overexpression of the respective r-protein partner has been previously observed for other r-protein/chaperone pairs [32,35,38]. Moreover, we observed that cells simultaneously lacking Acl4 and Rpl4a are substantially sicker than the individual single mutants (Fig 7B). Third, by binding to the long internal loop of Rpl4, which penetrates deeply into the rRNA core of the 60S subunit (see Fig 1), Acl4 may shield this highly basic region from engaging in illicit interactions with polyanions and, hence, aggregation prior to its final insertion into the cognate rRNA environment within nascent pre-60S subunits. Fourth, and in line with a protective function, Acl4 is required for the soluble expression of newly synthesized Rpl4 in yeast cells (Fig 7D).

Elegant experiments from the Görlich laboratory have demonstrated that r-proteins are prone to aggregation in the presence of polyanions, such as tRNA [18]. Notably, precipitation of selected r-proteins could be reversed by incubation with specific subsets of importins, thus establishing, besides their classical role as nuclear transport receptors, an anti-aggregation function for importins. It has therefore been postulated that, in order to be most efficiently protected, newly synthesized r-proteins should be immediately, possibly even co-translationally, shielded [18]. While it has not yet been revealed whether importins might already be recruited to nascent r-proteins, we have recently shown that four dedicated chaperones capture their specific r-protein partner in a co-translational manner [38]. In this study, we provide evidence that, likewise, Acl4 has the capacity to recognize Rpl4 as it is synthesized by the ribosome (Fig 8). Contrary to the other four cases, where the N-terminal regions of the r-proteins constitute the chaperone binding sites, Acl4 binds to an internal region, the C-terminal part of the long internal loop, of Rpl4. Further support for an early, cytoplasmic interaction is provided by the finding that Rpl4 is already bound by Acl4 within 5 min of its induced expression in an experiment combining the pulse-chase epitope labelling of Rpl4 with its affinity purification [45,50]. The necessity to protect Rpl4 as early as possible is also highlighted by the observation that it is susceptible to aggregation, like many other r-proteins, in the absence of the ribosome-associated chaperone systems (SSB/RAC and NAC) [22]. Collectively, this raises interesting questions about the coordination of the different co-translational processes, *e*.*g*.: how is a nascent r-protein, such as Rpl4, transferred from the general co-translational chaperones to its dedicated chaperone in order to ensure its productive synthesis as a soluble protein?

In agreement with cytoplasmic formation and nuclear co-import of the Acl4-Rpl4 complex, *in vitro* reconstitution experiments revealed that the importin Kap104 is capable of forming a stoichiometric trimeric complex with Acl4 and Rpl4 [45]. Notably, the association of the Acl4-Rpl4 heterodimer with Kap104 is dependent on the presence of Rpl4’s C-terminal extension, which harbours a complex NLS region consisting of two partially overlapping NLSs (Fig 2B) [45]. However, Rpl4 lacking the C-terminal extension is still targeted to the nucleus (Fig 2A), indicating that the other three identified NLSs are sufficient to mediate nuclear import. Since binding of Acl4 covers a region of Rpl4, encompassing amino acids 101–114, that is a critical nuclear-targeting determinant for two of these NLSs (Fig 2B and S4 Fig), it can be inferred that this NLS region should only be responsible for the import of free Rpl4 and not the Acl4-Rpl4 complex. Taken together and considering the quasi-essential nature of Acl4, we propose that the co-import of the Acl4-Rpl4 complex, mediated by binding of Kap104 to the C-terminal extension of Rpl4, represents the major import and pre-60S assembly pathway (Fig 9). However, it is very likely that other, yet to be determined importins may also recognize the complex NLS region within Rpl4’s C-terminal extension since its nuclear localization is not abolished in *kap104-16* mutant cells (S11 Fig). Given that yeast cells still grow, albeit at a very slow rate, in the absence of Acl4 (Fig 4D) [45], we suggest the existence of alternative, Acl4-independent import and pre-60S assembly routes for Rpl4 (Fig 9). In these minor import pathways, nuclear transport of Rpl4 is likely promoted by importin binding to one of the two internal or the C-terminally located NLS regions. Irrespective of the import route, Ran-GTP binding to the importin will release free or Acl4-bound Rpl4, which can then be incorporated into early pre-60S particles. Additionally, it is also possible that Rpl4, imported *via* an Acl4-independent route, will encounter and be transferred to an Acl4 molecule in the nucleoplasm. In this scenario, Acl4 would act as an escortin, as recently proposed to be the role of the Rps26 chaperone Tsr2 [33], connecting the nuclear import of Rpl4 to its fail-safe deposition on pre-60S subunits. Finally, it has to be assumed that most Acl4 molecules, in order to sustain the enormous demand for newly synthesized and assembly-competent Rpl4, should travel back, after pre-60S delivery of Rpl4, from the nucleus to the cytoplasm. However, it remains to be determined whether translocation of Acl4 across the NPC occurs by facilitated diffusion or relies on an active export mechanism.

Contrary to the result obtained by the Woolford laboratory [48], our study, as well as the one from the Hurt laboratory [45], clearly revealed that the eukaryote-specific extension of Rpl4 harbours an essential function (Fig 1C). Due to the importance of the eukaryote-specific C-terminal extension in coupling Acl4 release with the incorporation of Rpl4 into nascent pre-60S subunits, it was proposed that this region delivers the Acl4-Rpl4 complex to the pre-ribosomal particle by contacting co-evolved, eukaryote-specific sites [45]. Given that the efficient assembly of Rpl4 relies on critical hydrophobic contacts between residues of the C-terminal extension (*e*.*g*.: Ile289, Ile290, and Ile295) with Rpl18, a hierarchical assembly model has been put forward [45]. Accordingly, assembly of Rpl18 would precede and mediate, by contributing to the recruitment of the eukaryote-specific C-terminal extension, the initial docking of Rpl4, which would then be followed by the insertion of the long internal loop and the concomitant release of Acl4 [45]. However, several lines of evidence indicate that a complete understanding of the order of the assembly events will require further clarification. Contrary to the above-described contribution of certain hydrophobic residues of Rpl4 to its pre-60S incorporation and release from Acl4, Rpl4 gets, while only showing a moderate increase in Acl4 association, efficiently assembled into mature 60S subunits in cells expressing an Rpl18 variant (L32E, V129D) with mutations in the Rpl4 interaction surface [45]. Moreover, our C-terminal deletion analysis revealed that interactions of Rpl4, besides the contacts with Rpl18, with Rpl7 and ES7^L^, involving the middle part of the C-terminal extension (amino acids 302–332), are required for optimal cell growth and production of 60S subunits (Fig 1C and S3 Fig). Nevertheless, these mutant Rpl4 proteins (N325 and N301 constructs) get, in the presence of wild-type Rpl4, incorporated into mature 60S subunits and translating ribosomes (Fig 3B). More strikingly even, we observed in the same experimental setting that Rpl4 lacking the complete C-terminal extension (N264 construct) can be assembled, albeit less efficiently, into mature 60S subunits (Fig 3B); thus, indicating the possibility of an independent pre-60S assembly of the universally conserved part of Rpl4. However, the assembly of the globular domain and C-terminal extension seems to be tightly interconnected, as suggested by our observation that their separate expression confers growth to yeast cells and leads to the stable incorporation of both protein fragments into mature 60S subunits (S12 Fig). Further, given that the globular domain and the N-terminal part of the long internal loop of Rpl4 engage in a significant number of interactions with LSU rRNA domain I, while Rpl18 almost exclusively forms polar contacts with rRNA domain II, the initial association of Rpl4 with nascent pre-rRNA may occur prior to Rpl18 recruitment. Taken together, an alternative model for Rpl4 assembly, involving a series of interconnected steps, may be envisaged: (*i*) initial docking of Rpl4 is established by contacts of the globular domain and parts of the long internal loop with rRNA domain I; (*ii*) the C-terminal extension of Rpl4 facilitates Rpl18 and Rpl7 recruitment, thereby enabling formation of the eukaryote-specific interaction network and, thus, fortifying Rpl4 association and promoting correct pre-rRNA folding within this pre-60S region; (*iii*) either concomitantly or subsequently, the long internal loop of Rpl4 gets completely inserted into the rRNA core of pre-60S subunits, thereby leading to the dissociation of Acl4 from its Rpl4 binding site. These highly complex assembly events, coupling stable Rpl4 incorporation with Acl4 release, are expected to occur very fast since Acl4 is not detectably associated with pre-60S particles (Fig 4C and 4F). Moreover, the cellular Acl4 levels seem to influence the efficiency of Rpl4 association with pre-60S subunits, by affecting the equilibrium between Acl4-bound and pre-60S-associated Rpl4, since overexpression of Acl4 confers a strong slow-growth phenotype to wild-type cells (S13 Fig); this observation also suggests that Acl4 does not actively promote Rpl4 assembly.

The combined data from the three studies also provide compelling evidence for a role of the essential long internal loop of Rpl4 during late pre-60S maturation events that are necessary for the productive assembly of export-competent pre-60S subunits. In contrast to depletion of Rpl4, which entails early pre-60S assembly defects (S8 Fig) [43,48], it was shown that Rpl4 depleted cells expressing an Rpl4 protein lacking its long internal loop display a strong accumulation of the 7S pre-rRNA [48]. In line with such a requirement during late pre-rRNA processing steps leading to the formation of mature 5.8S rRNA, purification, upon pulse-chase epitope labelling, of Rpl4 lacking the tip region (amino acids 63–87) of the long internal loop yielded late pre-60S ribosomes, as inferred by the co-enrichment of the export adaptor Nmd3 and the GTPases Nog1 and Lsg1 [45]. Consistently, Rpl4 lacking the long internal loop, when expressed in wild-type cells, confers a slow-growth phenotype and, as revealed by sucrose gradient fractionation, gets efficiently incorporated into pre-60S subunits (Fig 3A and 3B). While the other two studies did not investigate in which compartment these aberrant pre-60S subunits are stalled, our report notably reveals, as suggested by the predominant nuclear localization of Rpl4 lacking the long internal loop (Fig 2A and 2B), that maturation of pre-60S particles is blocked at a nuclear step. Future experiments will be required to better define the specific step at which assembly of these pre-60S subunits is halted and how the long internal loop of Rpl4 promotes progression of late pre-60S maturation.

An interesting, yet puzzling observation is that the eukaryote-specific Acl4, despite its almost essential function in yeast, is not conserved in all evolutionary more advanced classes, including mammals, and also conspicuously absent from certain early evolutionary branches; thus, indicating that other proteins may relatively easily replace Acl4. Comparison of the Acl4 binding site within the long internal loop of Rpl4 between archaea and yeast reveals that this region, which has only limited sequence similarity, notably displays eye-catching differences with respect to the electrostatic surface properties (S14 Fig). This observation suggests that Acl4 might have arisen due to the necessity to shield the more positively charged, eukaryotic surface during transport of Rpl4 to its nuclear pre-60S assembly site. Given that certain importins (the importin-β/importin-7 heterodimer and importin-9) were shown to counteract the aggregation of mammalian rpL4 in the presence of polyanions [18], it is reasonable to speculate that these importins may fulfil a dual role as dedicated chaperone and transport receptor of rpL4 in mammalian cells.

In conclusion, our study has identified the previously uncharacterized Acl4 as a dedicated chaperone of the 60S subunit r-protein Rpl4 and has revealed that recognition may already occur during the cytoplasmic synthesis of Rpl4. Our findings underscore the necessity to protect r-proteins on their path to their ribosomal incorporation site and further validate co-translational capturing of r-proteins by dedicated chaperones as an advantageous and prevalently used concept to efficiently fulfil this task. Clearly, future work will be required to decipher the molecular details of the Acl4-Rpl4 interaction and to unveil the precise mechanisms that promote the stable assembly of Rpl4 into pre-60S subunits.

## Methods

### Yeast strains, genetic methods and plasmids

The *S*. *cerevisiae* strains used in this study are listed in Supporting S1 Table; all strains, unless otherwise specified, are derivatives of W303 [51]. For yeast two-hybrid analyses the reporter strain PJ69-4A was used [52]. Deletion disruption and C-terminal tagging at the genomic locus were performed as described [53,54]. Preparation of media, yeast transformation, and genetic manipulations were done according to established procedures. For the experiments involving induction of expression by addition of copper sulfate, media were prepared with copper-free yeast nitrogen base (FORMEDIUM). All recombinant DNA techniques were according to established procedures using *Escherichia coli* DH5α for cloning and plasmid propagation. Codon-optimized (for *E*. *coli* expression) *C*. *thermophilum ct*ACL4 and *ct*RPL4 genes were generated by custom DNA synthesis (Eurofins). All cloned DNA fragments generated by PCR amplification were verified by sequencing. More information on the plasmids, which are listed in Supporting S2 Table, is available upon request.

### Yeast two-hybrid (Y2H) interaction analysis

For Y2H-interaction assays, plasmids expressing bait proteins, fused to the Gal4 DNA-binding domain (G4BD), and prey proteins, fused to the Gal4 activation domain (G4AD), were co-transformed into reporter strain PJ69-4A. Y2H interactions were documented by spotting representative transformants in 10-fold serial dilution steps onto SC-Trp-Leu, SC-Trp-Leu-His (*HIS3* reporter), and SC-Trp-Leu-Ade (*ADE2* reporter) plates, which were incubated for 3 d at 30°C. Growth on SC-Trp-Leu-His plates is indicative of a weak/moderate interaction, whereas only relatively strong interactions permit growth on SC-Trp-Leu-Ade plates.

### Fluorescence microscopy

Live yeast cells were imaged by fluorescence microscopy using an Olympus BX54 microscope. Nop58-yEmCherry, expressed from the genomic locus under the control of the cognate promoter, was used as a nucleolar marker. The Image J software was used to process the images.

### Tandem-affinity purification and *in vitro* binding assays

Cells expressing Acl4-TAP and NTAP-Rpl4 were grown at 23°C in 4 l YPD medium to an optical density (OD_600_) of 2. Wild-type cells expressing NTAP-Rpl4a.N264 and NTAP-Rpl4a.N291 from plasmid were grown at 30°C in 4 l SC-Leu medium to an OD_600_ of 1.5. Cell extracts were obtained by glass bead lysis with a Pulverisette (Fritsch). Tandem-affinity purifications were performed in a buffer containing 50 mM Tris-HCl pH 7.5, 100 mM NaCl, 1.5 mM MgCl_2_, 5% glycerol, and 0.1% NP-40 as described [55]. The EGTA eluates were precipitated by the addition of TCA to a final concentration of 10% and, after an acetone wash, dissolved in 80 μl of 3x SDS sample buffer. Protein samples were separated on NuPAGE 4–12% Bis-Tris 12-well gels (Novex), run in 1x MES SDS running buffer, and subsequently stained with Brilliant Blue G Colloidal Coomassie (Sigma). The identity of the proteins contained in Coomassie-stained bands was determined by mass spectrometric analysis of peptides obtained by digestion with trypsin.

For *in vitro* binding assays between Rpl4-(His)_6_ and Acl4-Flag or between *ct*Rpl4-(His)_6_ and *ct*Acl4, proteins were co-expressed from pETDuet-1 (Novagen) in Rosetta(DE3) (Novagen) or BL21(DE3) (Novagen) *E*. *coli* cells, respectively. Cells were grown in 200 ml of lysogeny broth (LB) medium at 37°C and protein expression was induced at an OD_600_ of around 0.6 to 0.8 by the addition of IPTG to a final concentration of 0.5 mM. After 3 h of growth at 30°C, cells were harvested and stored at -80°C. Cells were resuspended in 25 ml lysis buffer (50 mM Tris-HCl pH 7.5, 200 mM NaCl, 1.5 mM MgCl_2_, 5% glycerol) and lysed with a M-110L Microfluidizer (Microfluidics). The lysate (30 ml volume) was adjusted by the addition of 300 μl 10% NP-40 to 0.1% NP-40 (note that from here onwards all buffers contained 0.1% NP-40). An aliquot of 100 μl of total extract (sample T) was taken and mixed with 100 μl of 6x loading buffer. The total extract was then centrifuged at 4°C for 20 min at 14’000 rpm. The soluble extract was transferred to a 50 ml Falcon tube and, as above, an aliquot of 100 μl of soluble extract (sample S) was taken and mixed with 100 μl of 6x loading buffer. The insoluble pellet fraction (sample P) was resuspended in 3 ml of lysis buffer and 10 μl thereof were mixed with 90 μl of lysis buffer and 100 μl of 6x loading buffer. The soluble extract (30 ml) was adjusted to 15 mM imidazole by adding 180 μl 2.5 M imidazole pH 8. Upon addition of 250 μl of Ni-NTA Agarose slurry (Qiagen), samples were incubated for 2 h on a turning wheel at 4°C. Then, the Ni-NTA Agarose beads were pelleted by centrifugation at 4°C for 2 min at 1’800 rpm, resuspended in 2 ml of lysis buffer, and transferred to a 2 ml Eppendorf tube. The Ni-NTA Agarose beads were first washed five times with 1 ml of lysis buffer containing 15 mM imidazole and then two times for 5 min, by rotation on a turning wheel at 4°C, with 1 ml lysis buffer containing 50 mM imidazole. Elution of bound proteins was carried out by incubation of the Ni-NTA Agarose beads with 1 ml of lysis buffer containing 500 mM imidazole for 5 min on a turning wheel at 4°C. The eluate (sample E) was transferred to a 1.5 ml Eppendorf tube and 100 μl thereof were mixed with 100 μl of 6x loading buffer. Protein samples (5 μl of samples T, P, S, and E) were separated on NuPAGE 4–12% Bis-Tris 15-well gels (Novex), run in 1x MES SDS running buffer, and subsequently stained with Brilliant Blue G Colloidal Coomassie (Sigma). For Western analysis, appropriate dilutions of the above samples were separated on Bolt 4–12% Bis-Tris Plus 15-well gels (Novex), run in 1x MES SDS running buffer, and proteins were subsequently blotted onto nitrocellulose membranes (GE Healthcare).

### Sucrose gradient analysis and fractionation

Cell extracts for polysome profile analyses were prepared as previously described [56] and eight A_260_ units were layered onto 10–50% sucrose gradients that were centrifuged at 38’000 rpm in a Sorvall TH-641 rotor at 4°C for 2 h 45 min. Sucrose gradients were analysed using an ISCO UA-6 system with continuous monitoring at A_254_. For the fractionation experiments, five A_260_ units were subjected to sucrose gradient centrifugation for 2h 45 min and 20 fractions of around 500 μl were collected and processed as described [57]. Precipitated proteins were resuspended in 50 μl 3x sample buffer and 5 μl each fraction was separated on NuPAGE 4–12% Bis-Tris 26-well gels (Novex), run in 1x MES or 1x MOPS SDS running buffer, and subsequently analyzed by Western blotting. As an input control, 0.05 A_260_ units of total cell extract was run alongside the fractions.

### Preparation of total yeast protein extracts and western analysis

Total yeast protein extracts were prepared as previously described [58]. Cultures were grown to an OD_600_ of around 0.8 and protein extracts were prepared from an equivalent of one OD_600_ of cells. Western blot analysis was carried out according to standard protocols. The following primary antibodies were used in this study: mouse monoclonal anti-FLAG (1:2’000–1:10’000; Sigma), anti-GFP (1:2’000; Roche), anti-HA (1:3’000; BAbCO), anti-His_6_ (1:500; Roche), and anti-Rpl3 (1:5’000; J. Warner, Albert Einstein College of Medicine, New York); rabbit polyclonal anti-Adh1 (1:50’000; obtained from the laboratory of C. De Virgilio, University of Fribourg), anti-CBP (1:15’000; Open Biosystems), anti-Rpl5 (1:5’000; S.R. Valentini, São Paulo State University, Araraquara), and anti-Rps3 (1:20’000; M. Seedorf, ZMBH, University of Heidelberg, Heidelberg). Secondary goat anti-mouse or anti-rabbit horseradish peroxidase-conjugated antibodies (Bio-Rad) were used at a dilution of 1:10’000. For detection of TAP-tagged proteins, the Peroxidase-Anti-Peroxidase soluble complex was used at a dilution of 1:20’000 (Sigma). Immobilized protein-antibody complexes were visualized by using enhanced chemiluminescence detection kits (Amersham ECL, GE Healthcare; PicoDetect, Applichem; WesternBright Sirius, Advansta).

### RNA analyses

Total RNA was extracted from exponentially grown cells (10 OD_600_ units) by the acid-phenol method and equal amounts of total RNA (5 μg) were separated on 1.2% agarose gels containing 6% formaldehyde or on 7% polyacrylamide gels containing 8 M urea. Northern hybridization was performed as previously described [59], utilizing the following oligonucleotides as probes:

Probe b (18S) 5’-CATGGCTTAATCTTTGAGAC-3’

Probe c (D/A2) 5-GACTCTCCATCTCTTGTCTTCTTG-3’

Probe d (A2/A3) 5’-TGTTACCTCTGGGCCC-3’

Probe e (5.8S) 5’-TTTCGCTGCGTTCTTCATC-3’

Probe f (E/C2) 5’-GGCCAGCAATTTCAAGTTA-3’

Probe g (C1/C2) 5’-GAACATTGTTCGCCTAGA-3’

Probe h (25S) 5’-CTCCGCTTATTGATATGC-3’

Probe 5S 5’-GGTCACCCACTACACTACTCGG-3’

The radioactive signals on the hybridized membranes were revealed using the Typhoon FLA 9400 imaging system and the supplied software (GE Healthcare).

For the determination of the rRNA composition of ribosomal particles, GFP-tagged Rpl4a was precipitated by a one-step GFP-Trap_A procedure that was slightly modified from the one suggested in the manufacturer’s instructions (ChromoTek). Briefly, wild-type cells expressing untagged Rpl4a (negative control) or N-terminally yEGFP-tagged Rpl4a or Rpl4a.N264 from plasmid were grown in 200 ml SC-Leu medium to an OD_600_ of 0.8. Cells were then washed twice with ice-cold water and finally resuspended in 500 μl of ice-cold lysis buffer (20 mM Tris-HCl pH 8.0, 5 mM Magnesium acetate, 200 mM KCl, 0.2% Triton X-100) supplemented with 1 mM DTT and containing a protease inhibitor cocktail (Complete, Roche). Cells were disrupted with glass beads by vigorous vortexing at 4°C for 12 min. Lysates were clarified by centrifugation in a microfuge at the maximum speed (approximately 16’100x g) for 15 min at 4°C. To each of the resulting total cell extracts, 30 μl of GFP-Trap_A beads, equilibrated with the same buffer, were added and the mixture was incubated for 1 h 30 min at 4°C with end-over-end tube rotation. After incubation, the beads were extensively washed seven times with 1 ml of the same buffer at 4°C and finally collected. RNA was extracted from the beads and the total cell extracts as previously described [60], and the extracted RNA was analysed by Northern blotting as above.

### *In vivo* ribosomal protein solubility assay

The *Δacl4* mutant cells, either containing empty vector or a centromeric plasmid expressing Acl4 from the *ADH1* promoter, were grown in a volume of 100 ml to an OD_600_ of around 0.7 and expression of C-terminally 2xHA-tagged Rpl4a was induced for 20 min from the *CUP1* promoter with 500 μM copper sulfate. After harvesting, cells were lysed with glass beads in a buffer containing 50 mM Tris-HCl pH 7.5, 100 mM NaCl, 1.5 mM MgCl_2_, 5% glycerol, and 0.1% NP-40 and cell extracts were centrifuged for 3 min at 3’000 rpm. Then, total cell extracts, 10 A_260_ units in a final volume of 500 μl, were subjected to centrifugation at 200’000 g for 1 h. Pellets were resuspended in 100 μl lysis buffer and equal amounts of the total extracts (T), soluble extracts (S), and pellet fractions (P) were analyzed by SDS-PAGE and Western blotting using an anti-HA antibody.

### Determination of co-translational capturing by qRT-PCR

Co-translational association of Syo1-FTpA and Acl4-TAP with nascent r-proteins was assessed by IgG-Sepharose pull-down and real-time quantitative reverse transcription PCR (real-time qRT-PCR) as previously described [38]. Oligonucleotide pairs for the specific amplification of DNA fragments, corresponding to the *RPL3* and *RPL5* mRNA, from the input cDNAs, obtained from total RNA or chaperone-associated RNA, have been previously described [38]. The following oligonucleotide pairs were used for the specific amplification of DNA fragments corresponding to the *RPL4* and *RPL11* mRNAs:

RPL4-I-forward 5’-ACCTCCGCTGAATCCTGGGGT-3’

RPL4-I-reverse 5’-ACCGGTACCACCACCACCAA-3’ (amplicon size 72 bp)

RPL11-I-forward 5’-ACACTGTCAGAACTTTCGGT-3’

RPL11-I-reverse 5’-TTTCTTCAGCCTTTGGACCT-3’ (amplicon size 81 bp)

### Sequence alignments, secondary structure prediction and analysis of 3D structures

Multiple sequence alignments of orthologous proteins were generated in the ClustalW output format with T-Coffee using the default settings of the EBI website interface [61]. Secondary structure prediction was performed with the PSIPRED v3.3 prediction method available at the PSIPRED website interface [62]. Potential tetratrico peptide repeats within Acl4 were identified by using the TPRpred website interface [63], in combination with secondary structure prediction, multiple sequence alignments, and visual inspection of the occurrence of TPR consensus residues [46,47]. To identify orthologues of *S*. *cerevisiae* Acl4, the sequence of the protein YD161_YEAST was searched for orthologues against the OMA database for orthology prediction (http://omabrowser.org/cgi-bin/gateway.pl?f=DisplayEntry&p1=YD161_YEAST) [64]. OMA identified 1:1 orthologues in 23 species in the first group, with orthologues in fungi, parasites, and fishes. A total of 97 orthologous sequences from eight groups (OMA groups: 351324, 181749, 130365, 204390, 227596, 336561, 539094, and 370534), as well as their corresponding NCBI taxid, were extracted from the orthoXML file. One group (273573 with six sequences) was excluded since it did not contain the characteristic TPR-repeat domain of this family. The sequences were aligned with MAFFT [65] and viewed with Jalview [66]. The taxids were pasted into the phyloT web server (http://phylot.biobyte.de) to generate the tree and forwarded to the iTOL web site to visualize the tree [67]. As shown by the multiple sequence alignment and the tree, the gene encoding Acl4 orthologues is found mainly in fungi, but some dispersed branches kept it (*e*.*g*.: some fishes, invertebrates, and parasites). Analysis and image preparation of three-dimensional structures, downloaded from the PDB archive, was carried out with the PyMOL (PyMOL Molecular Graphics System; http://pymol.org/) or Chimera (http://www.cgl.ucsf.edu/chimera) software. The coordinates of the following ribosome structures were used: *S*. *cerevisiae* 60S subunit (PDB 3U5H and 3U5I; [15]), *S*. *cerevisiae* 80S ribosome (PDB 4V88; [15]), and *Haloarcula marismortui* 50S subunit (PDB 4V9F; [68,69]. The representation of the electrostatic surface potential of the universally conserved part of yeast and archaeal Rpl4 was generated with Chimera by coulombic surface colouring.

## Supporting Information

S1 Table Yeast strains used in this study. (PDF)

S2 Table Plasmids used in this study. (PDF)

S1 Fig Pre-rRNA processing pathway in *S*. *cerevisiae*. **A,** Structure of an rDNA repeat unit. Each rDNA unit contains two independently transcribed elements. The long element is transcribed by RNA polymerase I into a polycistronic pre-rRNA, which contains the sequences of the mature 18S, 5.8S, and 25S rRNAs. The short element encodes the mature 5S rRNA and is transcribed by RNA polymerase III into a pre-5S rRNA. External, internal, and non-transcribed spacers (ETS, ITS, and NTS) are indicated. The mature rRNA species are shown as blue bars and the transcribed spacers as black lines; thinner, light gray lines represent the non-transcribed spacers. The transcription start sites are highlighted by red arrows. The processing sites and the location of the various probes used in this study are also indicated. **B,** Schematic representation of the pre-rRNA processing pathway. The long polycistronic pre-rRNA transcript can undergo either post- or co-transcriptional processing, leading to the generation of the 20S and 27SA_2_ pre-rRNAs, which are the pre-rRNA components of the first pre-40S and pre-60S particles. These two pre-rRNA species are then further processed into the mature 18S rRNA and the mature 5.8S and 25S rRNAs. For a recent review describing in detail the yeast pre-rRNA processing pathway and the involved endo- and exonucleases, see [6].
(PDF)

S2 Fig Absence of Rpl4a confers a mild slow-growth phenotype and a decrease in the levels of 60S subunits. **A,** Growth comparison of *Δrpl4a* and *Δrpl4b* null mutant cells. Cells of isogenic wild-type (WT), *Δrpl4a*, and *Δrpl4b* strains, as well as cells of a *Δrpl4a*/*Δrpl4b* strain complemented by plasmid-borne *RPL4A*, were spotted in 10-fold serial dilution steps onto a YPD plate, which was incubated for 1.5 d at 30°C. **B,** Comparison of the polysome profiles of *Δrpl4a* and *Δrpl4b* null mutant cells. The above strains were grown at 30°C in YPD medium and cell extracts were prepared under polysome-preserving conditions. Eight A_260_ units were resolved in 10–50% sucrose gradients and the absorption profiles were recorded by continuous monitoring at A_254_. Sedimentation is from left to right. The peaks of free 40S and 60S subunits, 80S free couples/monosomes, and polysomes are indicated. Half-mers are highlighted by arrowheads.
(PDF)

S3 Fig Partial deletion of Rpl4’s eukaryote-specific C-terminal extension affects growth and production of 60S subunits. **A,** *In vivo* phenotypes of cells expressing viable C-terminal deletion variants of Rpl4a. YCplac111-based plasmids expressing, under the control of the cognate promoter, full-length Rpl4a or the indicated C-terminal deletion variants were transformed into the *RPL4* shuffle strain YBP15. After plasmid shuffling on 5-FOA-containing plates, cells were restreaked on YPD plates and then spotted in 10-fold serial dilution steps onto YPD plates, which were incubated for the indicated times at 30°C and 37°C. **B,** Polysome profiles of cells expressing viable C-terminal deletion variants of Rpl4a. The above strains were grown at 30°C in YPD medium and cell extracts were prepared under polysome-preserving conditions. Eight A_260_ units were resolved in 10–50% sucrose gradients and the absorption profiles were recorded by continuous monitoring at A_254_. Sedimentation is from left to right. The peaks of free 40S and 60S subunits, 80S free couples/monosomes, and polysomes are indicated. Half-mers are highlighted by arrowheads. **C,** Subcellular localization of C-terminally truncated Rpl4a proteins. Plasmids expressing N-terminally yEGFP-tagged full-length Rpl4a and the indicated *rpl4a* truncation variants from the cognate *RPL4A* promoter were transformed into the *RPL4* shuffle strain YBP15. After plasmid shuffling on 5-FOA-containing plates, cells were grown in SC-Leu medium at 30°C and inspected by fluorescence microscopy.
(PDF)

S4 Fig Rpl4 harbours five distinct nuclear localization signals. **A,** Definition of the borders of the NLSs within Rpl4a. Plasmids expressing, under the transcriptional control of the *ADH1* promoter, the (GA)_5_-3xyEGFP control protein or the indicated Rpl4a fragments fused, *via* a (GA)_5_-linker, to a C-terminal 3xyEGFP were transformed into a wild-type strain expressing the nucleolar marker protein Nop58-yEmCherry from the genomic locus. Transformed cells were grown in SC-Leu medium at 30°C and inspected by fluorescence microscopy. **B,** Representation of the five NLSs of Rpl4a. The sequences and the position of the minimal NLSs within Rpl4a are indicated. The colour code to indicate the different features of Rpl4a is as in Fig 1C.
(PDF)

S5 Fig Overexpression of C-terminally truncated Rpl4a variants confers a dominant slow-growth phenotype. Empty vector and YCplac111-based plasmids expressing, under the control of the inducible *GAL1-10* promoter, full-length Rpl4a or the indicated C-terminal deletion variants were transformed into the haploid wild-type strain YDK11-5A. Transformants were restreaked on SC-Leu plates and cells were then spotted in 10-fold serial dilution steps onto SC-Leu (Glucose; Glc) and SGal-Leu (Galactose; Gal) plates, which were incubated for the indicated times at 30°C.
(PDF)

S6 Fig Rpl4a lacking the eukaryote-specific C-terminal extension associates with early pre-60S particles. Wild-type cells containing plasmids expressing untagged Rpl4a (control) or N-terminally yEGFP-tagged Rpl4a (GFP-*RPL4A*) or Rpl4a.N264 (GFP-*RPL4A*.*N264*) were grown in SC-Leu medium to an OD_600_ of 0.8. Immunoprecipitation was carried out by incubation of cell extracts with GFP-Trap_A agarose beads. RNA was extracted from the beads and an aliquot of the total extracts. The isolated RNAs, corresponding to 1% of the total extracts (T) and 45% of the immunoprecipitates (IP), were separated on 1.2% agarose gels containing 6% formaldehyde (upper panel) or on 7% polyacrylamide gels containing 8 M urea (lower panel), transferred onto a nylon membrane, and hybridized with the indicated probes (see S1 Fig for their location within the 35S pre-rRNA).
(PDF)

S7 Fig Assessment of the functionality of plasmid-encoded GFP- and TAP-tagged Rpl4a and genomically expressed Acl4-GFP and Acl4-TAP. **A,** Growth phenotype of cells expressing GFP- and TAP-tagged Rpl4a variants. YCplac111-based plasmids harbouring the genes encoding, under the transcriptional control of the cognate promoter, C-terminally yEGFP-tagged Rpl4a (*RPL4A*-(GA)_5_-yEGFP), N-terminally yEGFP-tagged Rpl4a (yEGFP-*RPL4A*), or N-terminally TAP/Flag-tagged Rpl4a (NTAPF-*RPL4A*) were transformed into the *RPL4* shuffle strain YBP15. After plasmid shuffling on 5-FOA-containing plates, cells were restreaked on YPD plates and then spotted in 10-fold serial dilution steps onto YPD plates, which were incubated for the indicated times at 24°C, 30°C, and 37°C. **B,** Growth phenotype of cells expressing Acl4-GFP and Acl4-TAP from the genomic locus. Cells of the *ACL4*-GFP and *ACL4*-TAP strains, as well as cells of isogenic wild-type strains (*ACL4*), originating from spore clones of the same tetrads as the *ACL4*-GFP and *ACL4*-TAP strains, were spotted in 10-fold serial dilution steps onto YPD plates, which were incubated for the indicated times at 16°C, 24°C, 30°C, and 37°C.
(PDF)

S8 Fig Absence of Acl4 leads to a reduction in 27SB and 7S pre-rRNAs levels. **A,** Genetic depletion of Rpl4 abolishes growth of yeast cells. The *RPL4* shuffle strain YBP15 was transformed with plasmids expressing untagged or N-terminally 2xHA-tagged Rpl4 from either the cognate *RPL4A* promoter or the *GAL1-10* promoter. After plasmid shuffling on SGal plates containing 5-FOA, cells were restreaked on YPGal plates and then spotted in 10-fold serial dilution steps onto YPGal and YPD plates, which were incubated for 3 d at 30°C. **B,** Time-course of Rpl4a depletion. Cells expressing N-terminally 2xHA-tagged Rpl4a from the galactose-inducible *GAL1-10* promoter, as the sole cellular Rpl4 source, were first grown at 30°C in YPGal medium and then shifted to YPD medium. Cell extracts were prepared from samples harvested after the indicated times of growth in YPD medium (hours in glucose) and subjected to Western blot analysis using an anti-HA antibody. Note that the lanes of the different time points have been cut out from a Western blot image containing additional time points. **C,** Depletion of Rpl4 results in a reduced production of 60S subunits. The above strain (P*GAL*-HA-*RPL4A*) was grown at 30°C in YPGal medium and then shifted to YPD medium. Cell extracts were prepared, from cells grown in YPGal medium or from samples harvested at the indicated times of growth in YPD medium, under polysome-preserving conditions. Eight A_260_ units were resolved in 10–50% sucrose gradients and the absorption profiles were recorded by continuous monitoring at A_254_. Sedimentation is from left to right. The peaks of free 40S and 60S subunits, 80S free couples/monosomes, and polysomes are indicated. Half-mers are highlighted by arrowheads. **D,** Effects of Rpl4 depletion on steady-stated levels of pre-rRNAs and mature rRNAs. Strains expressing, as the sole Rpl4 source, untagged (*RPL4A*) or N-terminally 2xHA-tagged Rpl4a (HA-*RPL4A*) from the cognate *RPL4A* promoter or N-terminally 2xHA-tagged Rpl4a (P*GAL*-HA-*RPL4A*) from the *GAL1-10* promoter were first grown at 30°C in YPGal medium and then shifted for up to 6 h to YPD medium. Cells were harvested at the indicated times of growth in glucose and total RNA was extracted. Equal amounts of total RNA (5 μg) were separated on 1.2% agarose gels containing 6% formaldehyde (upper panel) or on 7% polyacrylamide gels containing 8 M urea (lower panel), transferred onto a nylon membrane, and hybridized with the indicated probes (see S1 Fig for their location within the 35S pre-rRNA). **E,** Effects of the absence of Acl4 on steady-stated levels of pre-rRNAs and mature rRNAs. Cells of two independent wild-type (WT) and *Δacl4* (Δ) strains were grown at 30°C in YPD medium. Total RNA was extracted and equal amounts (5 μg) were analyzed by Northern blotting as described above.
(PDF)

S9 Fig Amino acids 89–115 of *ct*Rpl4 are sufficient to mediate the *in vitro* interaction with *ct*Acl4. **A,** *C*. *thermophilum* Acl4 complements the growth defect of *Δacl4* null mutant cells. The *Δacl4* null mutant was transformed with YCplac111 (empty vector), YCplac111-*ACL4*, and pADH111-*ctACL4*. As a control, an isogenic wild-type strain was transformed with YCplac111. Transformants were restreaked and cells were spotted in 10-fold serial dilution steps onto SC-Leu plates, which were incubated for the indicated times at 24°C, 30°C, and 37°C. **B,** *In vitro* binding assay between *ct*Rpl4 and *ct*Acl4. The indicated C-terminally (His)_6_-tagged *ct*Rpl4 variants and full-length *ct*Acl4-Flag were co-expressed in *E*. *coli* and purified *via* Ni-affinity purification. Proteins were revealed by SDS-PAGE and Coomassie staining (top) or by Western blot analysis using anti-Flag (*ct*Acl4-Flag) and anti-His (*ct*Rpl4-(His)_6_ variants) antibodies (bottom). T, total extract (lane 1); P, pellet fraction (insoluble proteins, lane 2); S, soluble extract (lane 3); E, imidazole eluate (lane 4); M, molecular weight standard. Blue arrowheads highlight the bands corresponding to the different *ct*Rpl4-(His)_6_ variants used as baits for the purifications. Black arrowheads indicate the position of *ct*Acl4-Flag.
(PDF)

S10 Fig Growth phenotypes and expression levels of Rpl4 variants with alanine-block substitutions in the C-terminal part of the long internal loop. **A,** *In vivo* phenotypes of cells expressing Rpl4a variants containing non-overlapping, consecutive alanine substitutions within the C-terminal part of the long internal loop. The amino acid substitutions within each of the four alanine-block mutants (BI-mt, BII-mt, BIII-mt, and BIV-mt) are indicated in Fig 6A. YCplac111-based plasmids expressing, under the control of the cognate promoter, full-length Rpl4a or the indicated alanine-substitution mutants were transformed into the *RPL4* shuffle strain YBP15. After plasmid shuffling on 5-FOA-containing plates, cells were restreaked on YPD plates and then spotted in 10-fold serial dilution steps onto YPD plates, which were incubated for the indicated times at 23°C, 30°C and 37°C. **B,** Expression of Rpl4a alanine-substitution variants does not confer a growth defect to wild-type cells. Empty vector (YCplac111) and plasmid-borne wild-type *RPL4A* or the indicated alanine-substitution mutants, expressed under the control of the cognate promoter, were transformed into the haploid wild-type strain YDK11-5A. Transformants were restreaked and cells were spotted in 10-fold serial dilution steps onto SC-Leu plates, which were incubated for 2.5 d at 30°C. **C,** Expression levels of Rpl4a alanine-substitution variants. Empty vector (YCplac111) and plasmid-borne, N-terminally 2xHA-tagged wild-type *RPL4A* or the indicated alanine-substitution mutants, expressed under the control of the cognate promoter, were transformed into the haploid wild-type strain YDK11-5A. Expression levels were assessed by subjecting whole cell lysates to SDS-PAGE and Western analysis using anti-HA and anti-Adh1 (loading control) antibodies.
(PDF)

S11 Fig Kap104 is not required for nuclear accumulation of the C-terminal extension of Rpl4. Plasmids expressing, under the transcriptional control of the *ADH1* promoter, the C-terminal extension of Rpl4 (amino acids 263–362) or the PY-NLS region of Nab2 (amino acids 216–242) fused, *via* a (GA)_5_-linker, to a C-terminal 3xyEGFP were transformed into *KAP104* wild-type or *kap104-16* mutant cells. Transformed cells were grown at semi-permissive temperature (23°C) in SC-Leu medium and the localization was assessed by fluorescence microscopy after a shift for 40 min to the non-permissive temperature (37°C).
(PDF)

S12 Fig Yeast cells expressing separately Rpl4’s globular domain and C-terminal extension are viable. **A,** Separate expression of the universally conserved globular domain and the eukaryote-specific C-terminal extension results in intramolecular complementation of the *Δrpl4a*/*Δrpl4b* null mutant phenotype. The *RPL4* shuffle strain YBP15 was co-transformed with empty vector or plasmids expressing full-length Rpl4a or the C-terminal extension of Rpl4a (amino acids 263–362) and empty vector or a plasmid expressing the globular domain of Rpl4a (N264 construct; amino acids 1–264). These plasmids express full-length Rpl4a and its fragments from the cognate *RPL4A* promoter. Transformants were restreaked on SC-Leu-Trp plates and cells were spotted in 10-fold serial dilution steps onto SC-Leu-Trp plates and 5-FOA containing (+5-FOA) plates, which were incubated for the indicated times at 30°C. **B,** The separately expressed globular domain and C-terminal extension get incorporated into mature 60S subunits. The *RPL4* shuffle strain YBP15 was co-transformed with plasmids expressing separately the N-terminally 2xHA-tagged globular domain (N264) and the C-terminally yEGFP-tagged C-terminal extension (amino acids 263–362). After plasmid shuffling on 5-FOA containing plates, cells were grown at 30°C in YPD medium to an OD_600_ of around 0.8. Whole cell lysates were prepared under polysome-preserving conditions in the presence of cycloheximide and analyzed by sucrose gradient centrifugation and fractionation. Five A_260_ units were resolved in 10–50% sucrose gradients and the absorption profile was recorded by continuous monitoring at A_254_ (upper panel). Sedimentation is from left to right. The peaks of free 40S and 60S subunits, 80S free couples/monosomes, and polysomes are indicated. Half-mers are highlighted by arrowheads. Gradient fractions were subjected to Western blot analysis using anti-HA, anti-GFP, and anti-Rpl3 antibodies (lower panel).
(PDF)

S13 Fig Overexpression of Acl4 affects growth of wild-type cells. Empty vector and monocopy or multicopy plasmids expressing Acl4 under the transcriptional control of the inducible *GAL1-10* promoter were transformed into the haploid wild-type strain YDK11-5A. Transformants were restreaked on SC-Leu plates and cells were then spotted in 10-fold serial dilution steps onto SC-Leu (Glucose; Glc) and SGal-Leu (Galactose; Gal) plates, which were incubated for the indicated times at 30°C.
(PDF)

S14 Fig The C-terminal part of Rpl4’s long internal loop displays eukaryote-specific surface features. Comparison of the Acl4 binding site within the long internal loop of Rpl4 between yeast and archaea reveals that this region displays notable differences in its electrostatic surface properties. The structures of *S*. *cerevisiae* Rpl4 (amino acids 1–264) (upper panel) and full-length *H*. *marismortui* L4 (lower panel), extracted from PDB’s 4V88 and 4V9F, respectively, were generated in Chimera and are shown in dark gray as ribbon representation. Side chains of residues 88–114 of *S*. *cerevisiae* Rpl4 and residues 68–103 of *H*. *marismortui* L4 are shown and coloured by element. Coulombic surface colouring was applied to visualize, in a semi-transparent representation, the electrostatic surface properties. The following settings were used: Number of colours (default 3: red, white, blue), Range (-15 to 15 kcal/(mol·*e*)), Distance-dependent dielectric (true), Dielectric constant (default 4.0), and Distance from surface (default 1.4Å). The structures are shown in three different orientations. Landmark residues within the long internal loop and the main Acl4 interaction surface (amino acids 88–114), as well as the analogous archaeal surface, are indicated.
(PDF)
