# Supplementary material for: The Dedicated Chaperone Acl4 Escorts Ribosomal Protein Rpl4 to Its Nuclear Pre-60S Assembly Site
Source: PLoS Genet. 2015 Oct 8;11(10):e1005565. doi: 10.1371/journal.pgen.1005565 (PMC4598080; doi:10.1371/journal.pgen.1005565)
Supplement: S1 Table — (PDF) [file pgen.1005565.s001.pdf]

S1 Table. Yeast strains used in this study.

Name		Relevant genotype	Source
YBP15	*MAT*a	*rpl4a*::natNT2 *rpl4b*::HIS3MX4 *ade3*::kanMX4 pHT4467Δ-*RPL4A*	This study
YBP1	*MAT*a	*rpl4a*::natNT2	This study
YBP9	*MAT*a	*rpl4b*::natNT2	This study
YKL681	*MAT*a	*ACL4*-TAP::natNT2	This study
YKL689	*MAT*α	*ACL4*-TAP::HIS3MX4	This study
YKL693	*MAT*α	*ACL4*-GFP::HIS3MX4	This study
YKL757	*MAT*a	*ACL4*-GFP::HIS3MX4 *NOP58*-yEmCherry::natNT2 *ade3*::kanMX4	This study
YKL701	*MAT*a	*acl4*::HIS3MX4	This study
YKL703	*MAT*α	*acl4*::natNT2	This study
YBP92	*MAT*α	*ACL4 RPL4A RPL4B*	This study
YBP91	*MAT*a	*acl4*::natNT2 *RPL4A RPL4B*	This study
YBP97	*MAT*a	*ACL4 rpl4A*::HIS3MX4 *RPL4B*	This study
YBP98	*MAT*a	*acl4*::natNT2 *rpl4A*::HIS3MX4 *RPL4B*	This study
YBP89	*MAT*α	*ACL4 RPL4A rpl4B*::HIS3MX4	This study
YBP90	*MAT*a	*acl4*::natNT2 *RPL4A rpl4B*::HIS3MX4	This study
YKL500	*MAT*α	*NOP58*-yEmCherry::natNT2 *ade3*::kanMX4	This study
YDK11-5A	*MAT*α	*ade3*::kanMX4	Kressler *et al*. 1999
Y4047	*MAT*a	*SYO1*-FTpA::HIS3MX4	Kressler *et al*. 2012
Y4252	*MAT*α	*kap104*::natNT2 *ade3*::kanMX4 pRS316-*KAP104*	This study
PJ69-4A	*MAT*a	*trp1-901 leu2-3,112 ura3-52 his3-200 gal4Δ gal80Δ LYS2*::*GAL1-HIS3 GAL2-ADE2 met2*::*GAL7*-lacZ	James *et al*. 1996

Strains used in this study were derived from W303 (*MAT***a**/*MAT*α *ade2-1*/*ade2-1 his3-11,15*/*his3-11,15 leu2-3,112*/*leu2-3,112 trp1-1*/*trp1-1 ura3-1*/*ura3-1 can1-100*/*can1-100*). For yeast two-hybrid interaction assays, strain PJ69-4A was used.
