# Supplementary material for: The Dedicated Chaperone Acl4 Escorts Ribosomal Protein Rpl4 to Its Nuclear Pre-60S Assembly Site
Source: PLoS Genet. 2015 Oct 8;11(10):e1005565. doi: 10.1371/journal.pgen.1005565 (PMC4598080; doi:10.1371/journal.pgen.1005565)
Supplement: S2 Table — (PDF) [file pgen.1005565.s002.pdf]

S2 Table. *S. cerevisiae* and *E. coli* expression plasmids used in this study.*

Name	Relevant information	Source
YCplac111-P*RPL4A*-*RPL4A*	CEN, *LEU2*, P*RPL4A*, T*ADH1*	This study
pHT4467Δ-*RPL4A*-stop	CEN6 (instable), *URA3*, *ADE3*, P*RPL4A*, T*ADH1*	This study
pyEGFPGAC111-*RPL4A*	CEN, *LEU2*, P*RPL4A*, T*ADH1*<br>C-terminal $(GA)_5$-yEGFP	This study
pyEGFPN111-*RPL4A*	CEN, *LEU2*, P*RPL4A*, T*ADH1*, N-terminal yEGFP	This study
pADH111-*RPL4A*-$(GA)_5$-3xyEGFP	CEN, *LEU2*, P*ADH1*, T*ADH1*<br>C-terminal $(GA)_5$-3xyEGFP	This study
pNTAPF111-*RPL4A*	CEN, *LEU2*, P*RPL4A*, T*ADH1*<br>N-terminal protA-TEV-CBP-Flag tag	This study
pGAL111-*RPL4A*	CEN, *LEU2*, P*GAL1-10*, T*ADH1*	This study
pGAL111-HA-*RPL4A*	CEN, *LEU2*, P*GAL1-10*, T*ADH1*, N-terminal 2xHA	This study
pHAN111-*RPL4A*	CEN, *LEU2*, P*RPL4A*, T*ADH1*, N-terminal 2xHA	This study
pCUP111-*RPL4A*-2xHA	CEN, *LEU2*, P*CUP1*, T*ADH1*, C-terminal 2xHA	This study
pGAG4BDC22-*RPL4A*	CEN, *TRP1*, P*ADH1*, T*ADH1*, C-terminal $(GA)_5$-G4BD	This study
pG4ADHAN111-*RRB1*	CEN, *LEU2*, P*ADH1*, T*ADH1*, N-terminal G4AD	This study
YCplac111-*ACL4*	CEN, *LEU2*, P*ACL4*, T*ADH1*	This study
YEplac112-*ACL4*	2μ, *TRP1*, P*ACL4*, T*ADH1*	This study
pADH22-*ACL4*	CEN, *TRP1*, P*ADH1*, T*ADH1*	This study
pGAL22-*ACL4*	CEN, *TRP1*, P*GAL1-10*, T*ADH1*	This study
pGAL111-*ACL4*	CEN, *LEU2*, P*GAL1-10*, T*ADH1*	This study
pGAL181-*ACL4*	2μ, *LEU2*, P*GAL1-10*, T*ADH1*	This study
pGAG4ADC111-*ACL4*	CEN, *LEU2*, P*ADH1*, T*ADH1*, C-terminal $(GA)_5$-G4AD	This study
pADH111-*ctACL4*	CEN, *LEU2*, P*ADH1*, T*ADH1*	This study
YCplac22-*KAP104*	CEN, *TRP1*, P*KAP104*, T*KAP104*	This study
pRS314-*kap104-16*	CEN, *TRP1*, P*KAP104*, T*KAP104*	Aitchison *et al.* 1996
pADH111-*NAB2(216-242)*-$(GA)_5$-3xyEGFP	CEN, *LEU2*, P*ADH1*, T*ADH1*<br>C-terminal $(GA)_5$-3xyEGFP	This study
pFA6a-HIS3MX4	for genomic deletion disruption	Longtine *et al.* 1998
pFA6a-natNT2	for genomic deletion disruption	Janke *et al.* 2004
pFA6a-GFP-HIS3MX4	GFP(S65T), T*ADH1*, for genomic C-terminal tagging	Longtine *et al.* 1998
pFA6a-TAP-HIS3MX4	TAP, T*ADH1*; for genomic C-terminal tagging	Pausch *et al.* 2015
pFA6a-TAP-T*CYC1*-natNT2	TAP, T*CYC1*; for genomic C-terminal tagging	Kressler *et al.* 2008
pFA6a-yEmCherry-natNT2	yEmCherry; for genomic C-terminal tagging	This study
pETDuet-1/*RPL4A*-$(His)_6$--*ACL4*-Flag	amp^r, T7 promoter/*lac* operator	This study
pETDuet-1/*ctRPL4A*-$(His)_6$--*ctACL4*-Flag	amp^r, T7 promoter/*lac* operator	This study

*For simplicity, only the plasmids containing the respective wild-type genes are listed. The mutant variants thereof used in this study were cloned into the listed plasmids. P and T denote promoter and terminator, respectively.
