# Supplementary figures and images for: The Dedicated Chaperone Acl4 Escorts Ribosomal Protein Rpl4 to Its Nuclear Pre-60S Assembly Site
Source: PLoS Genet. 2015 Oct 8;11(10):e1005565. doi: 10.1371/journal.pgen.1005565 (PMC4598080; doi:10.1371/journal.pgen.1005565)

**Figure S1**

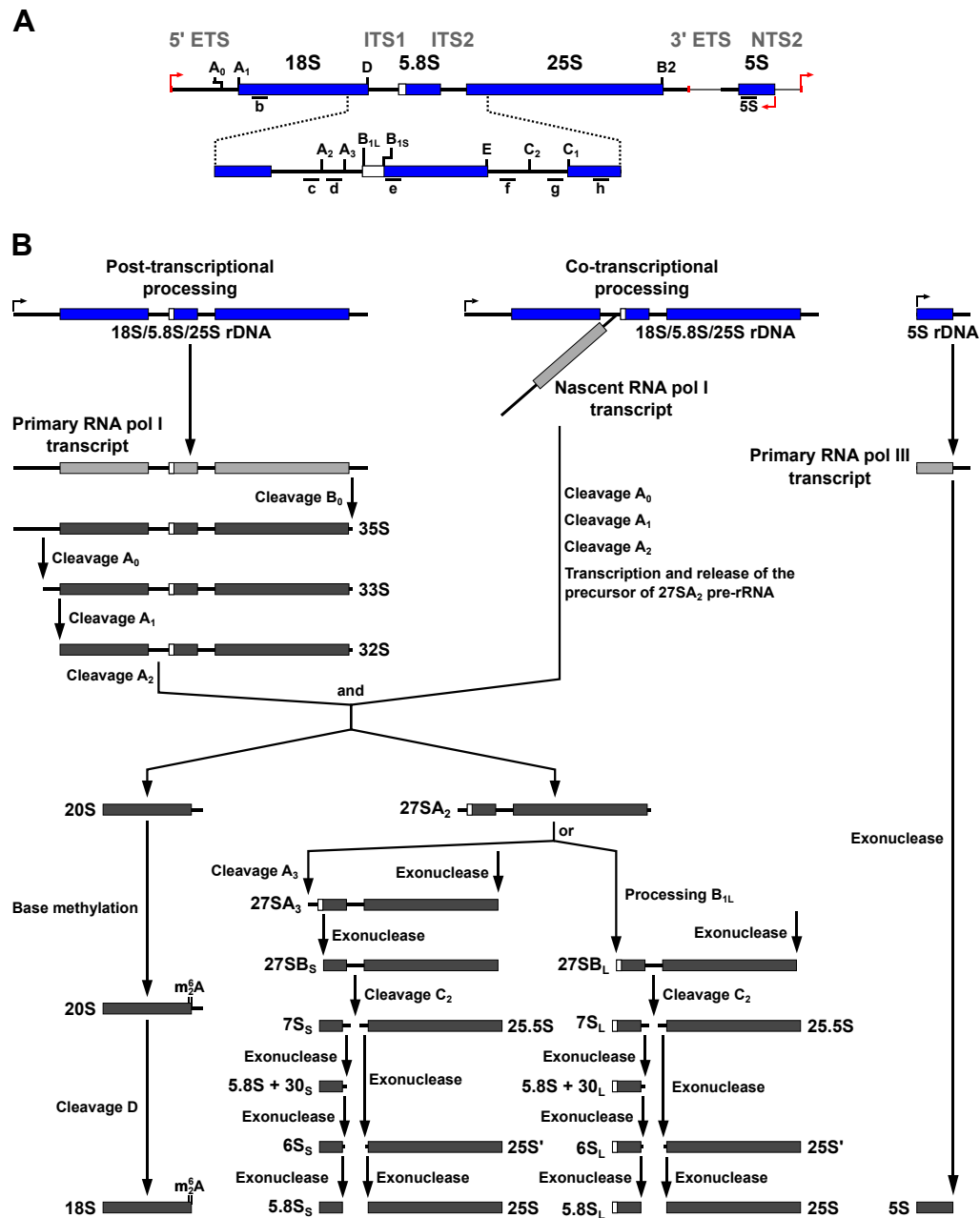

Supplement: S1 Fig — A, Structure of an rDNA repeat unit. Each rDNA unit contains two independently transcribed elements. The long element is transcribed by RNA polymerase I into a polycistronic pre-rRNA, which contains the sequences of the mature 18S, 5.8S, and 25S rRNAs. The short element encodes the mature 5S rRNA and is transcribed by RNA polymerase III into a pre-5S rRNA. External, internal, and non-transcribed spacers (ETS, ITS, and NTS) are indicated. The mature rRNA species are shown as blue bars and the transcribed spacers as black lines; thinner, light gray lines represent the non-transcribed spacers. The transcription start sites are highlighted by red arrows. The processing sites and the location of the various probes used in this study are also indicated. B, Schematic representation of the pre-rRNA processing pathway. The long polycistronic pre-rRNA transcript can undergo either post- or co-transcriptional processing, leading to the generation of the 20S and 27SA2 pre-rRNAs, which are the pre-rRNA components of the first pre-40S and pre-60S particles. These two pre-rRNA species are then further processed into the mature 18S rRNA and the mature 5.8S and 25S rRNAs. For a recent review describing in detail the yeast pre-rRNA processing pathway and the involved endo- and exonucleases, see [6]. (PDF) [file pgen.1005565.s003.pdf]

**Figure S2**

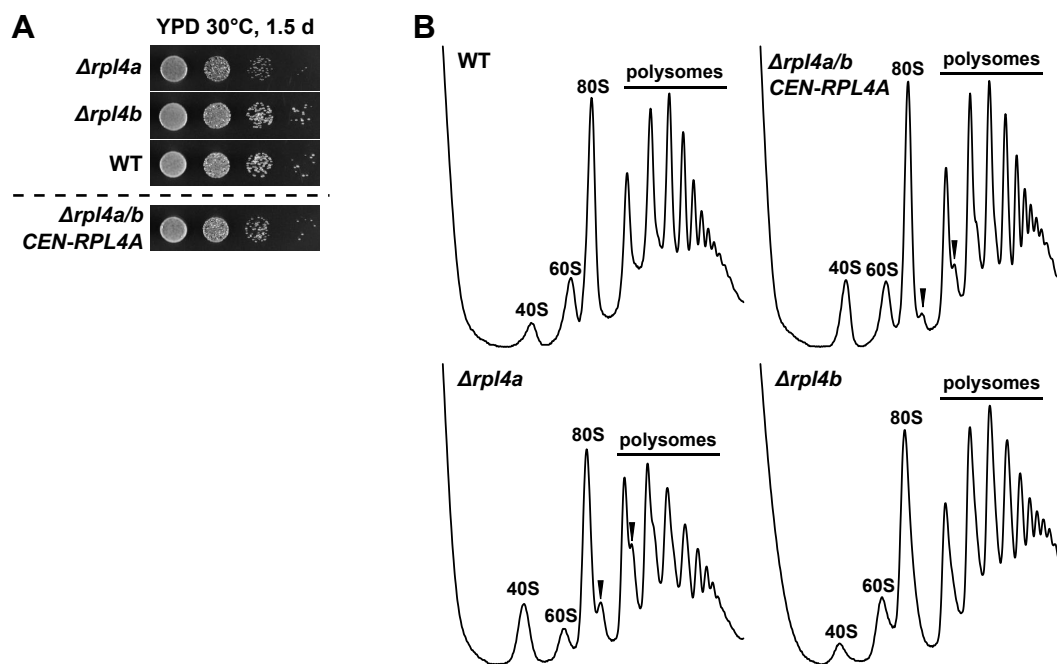

Supplement: S2 Fig — A, Growth comparison of Δrpl4a and Δrpl4b null mutant cells. Cells of isogenic wild-type (WT), Δrpl4a, and Δrpl4b strains, as well as cells of a Δrpl4a/Δrpl4b strain complemented by plasmid-borne RPL4A, were spotted in 10-fold serial dilution steps onto a YPD plate, which was incubated for 1.5 d at 30°C. B, Comparison of the polysome profiles of Δrpl4a and Δrpl4b null mutant cells. The above strains were grown at 30°C in YPD medium and cell extracts were prepared under polysome-preserving conditions. Eight A260 units were resolved in 10–50% sucrose gradients and the absorption profiles were recorded by continuous monitoring at A254. Sedimentation is from left to right. The peaks of free 40S and 60S subunits, 80S free couples/monosomes, and polysomes are indicated. Half-mers are highlighted by arrowheads. (PDF) [file pgen.1005565.s004.pdf]

**Figure S3**

**A**

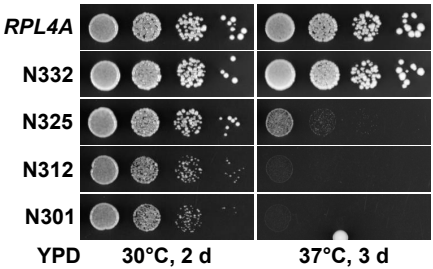

**C**

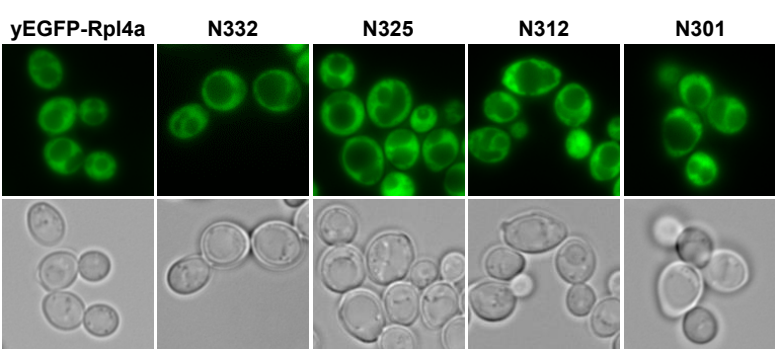

**B**

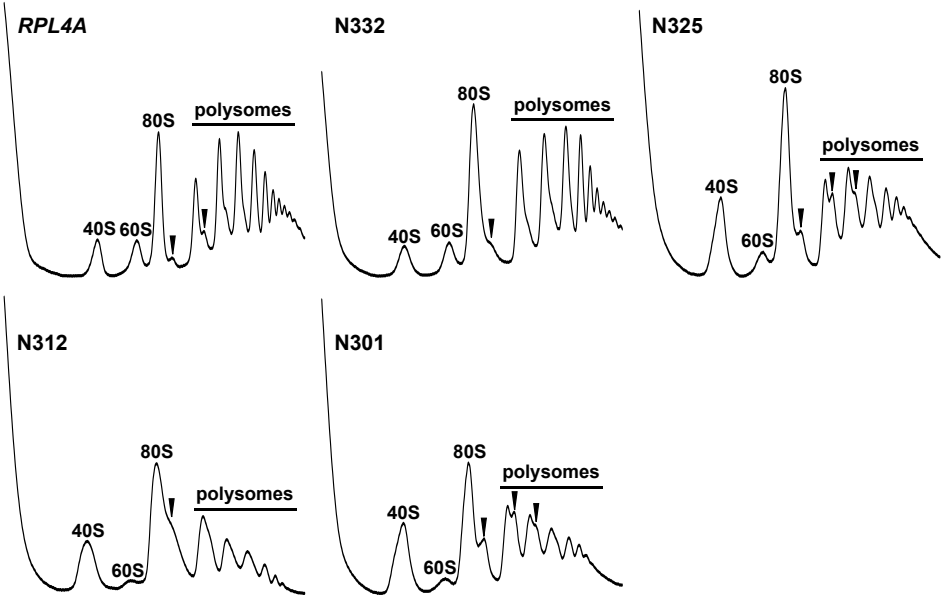

Supplement: S3 Fig — A, In vivo phenotypes of cells expressing viable C-terminal deletion variants of Rpl4a. YCplac111-based plasmids expressing, under the control of the cognate promoter, full-length Rpl4a or the indicated C-terminal deletion variants were transformed into the RPL4 shuffle strain YBP15. After plasmid shuffling on 5-FOA-containing plates, cells were restreaked on YPD plates and then spotted in 10-fold serial dilution steps onto YPD plates, which were incubated for the indicated times at 30°C and 37°C. B, Polysome profiles of cells expressing viable C-terminal deletion variants of Rpl4a. The above strains were grown at 30°C in YPD medium and cell extracts were prepared under polysome-preserving conditions. Eight A260 units were resolved in 10–50% sucrose gradients and the absorption profiles were recorded by continuous monitoring at A254. Sedimentation is from left to right. The peaks of free 40S and 60S subunits, 80S free couples/monosomes, and polysomes are indicated. Half-mers are highlighted by arrowheads. C, Subcellular localization of C-terminally truncated Rpl4a proteins. Plasmids expressing N-terminally yEGFP-tagged full-length Rpl4a and the indicated rpl4a truncation variants from the cognate RPL4A promoter were transformed into the RPL4 shuffle strain YBP15. After plasmid shuffling on 5-FOA-containing plates, cells were grown in SC-Leu medium at 30°C and inspected by fluorescence microscopy. (PDF) [file pgen.1005565.s005.pdf]

Figure S4

A

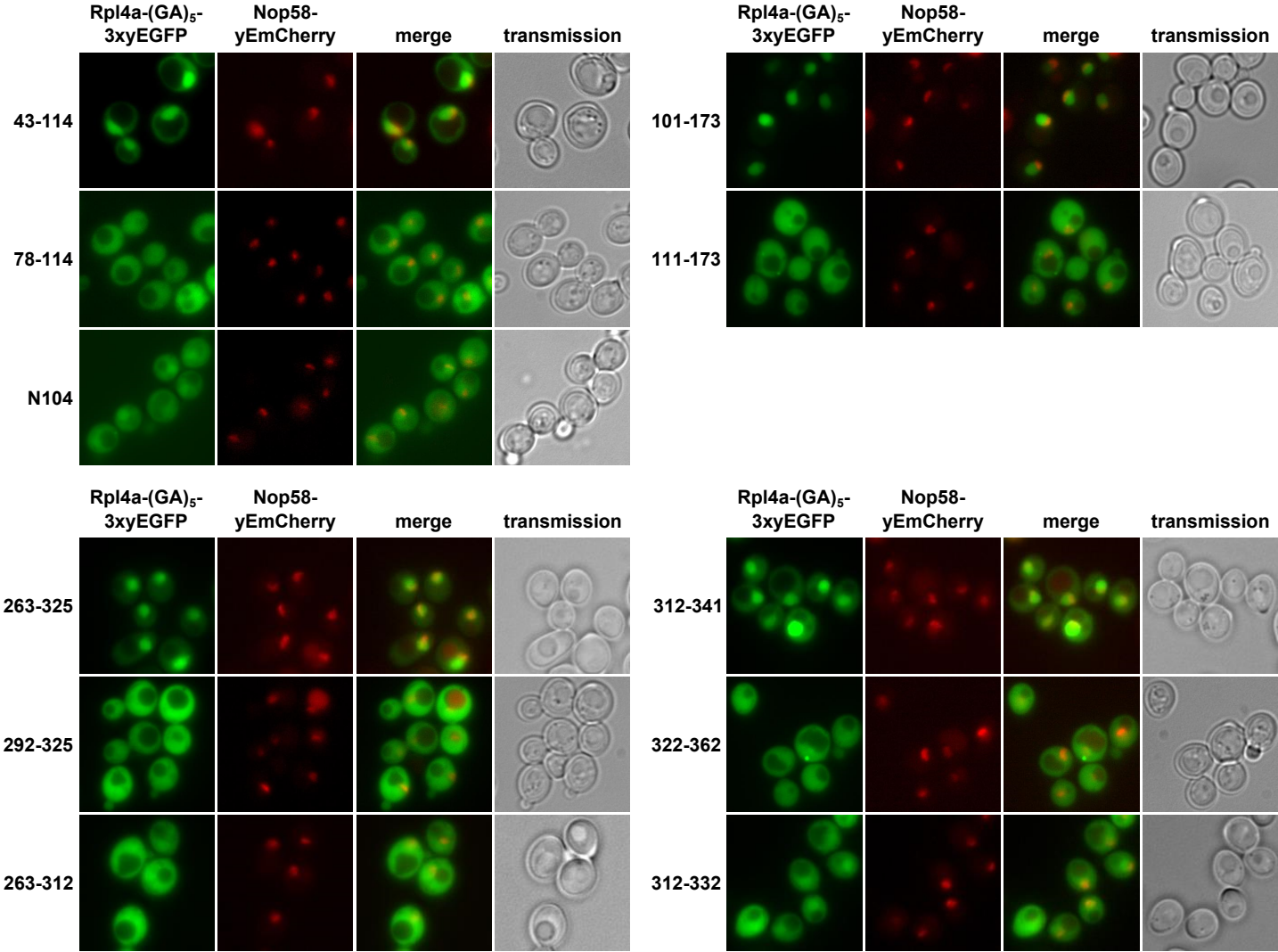

B

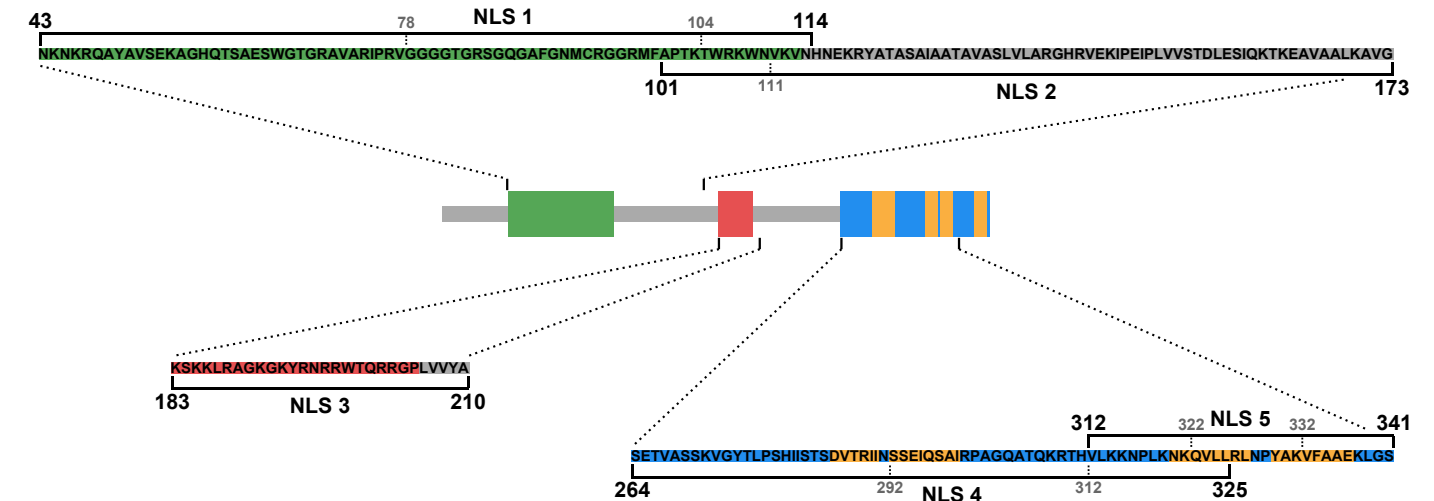

Supplement: S4 Fig — A, Definition of the borders of the NLSs within Rpl4a. Plasmids expressing, under the transcriptional control of the ADH1 promoter, the (GA)5-3xyEGFP control protein or the indicated Rpl4a fragments fused, via a (GA)5-linker, to a C-terminal 3xyEGFP were transformed into a wild-type strain expressing the nucleolar marker protein Nop58-yEmCherry from the genomic locus. Transformed cells were grown in SC-Leu medium at 30°C and inspected by fluorescence microscopy. B, Representation of the five NLSs of Rpl4a. The sequences and the position of the minimal NLSs within Rpl4a are indicated. The colour code to indicate the different features of Rpl4a is as in Fig 1C. (PDF) [file pgen.1005565.s006.pdf]

Figure S5

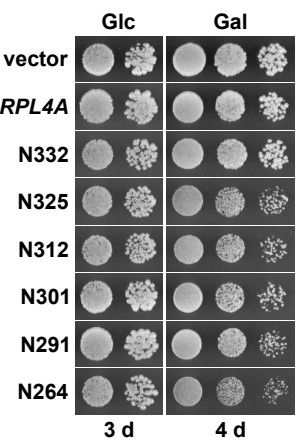

Supplement: S5 Fig — Empty vector and YCplac111-based plasmids expressing, under the control of the inducible GAL1-10 promoter, full-length Rpl4a or the indicated C-terminal deletion variants were transformed into the haploid wild-type strain YDK11-5A. Transformants were restreaked on SC-Leu plates and cells were then spotted in 10-fold serial dilution steps onto SC-Leu (Glucose; Glc) and SGal-Leu (Galactose; Gal) plates, which were incubated for the indicated times at 30°C. (PDF) [file pgen.1005565.s007.pdf]

## Figure S6

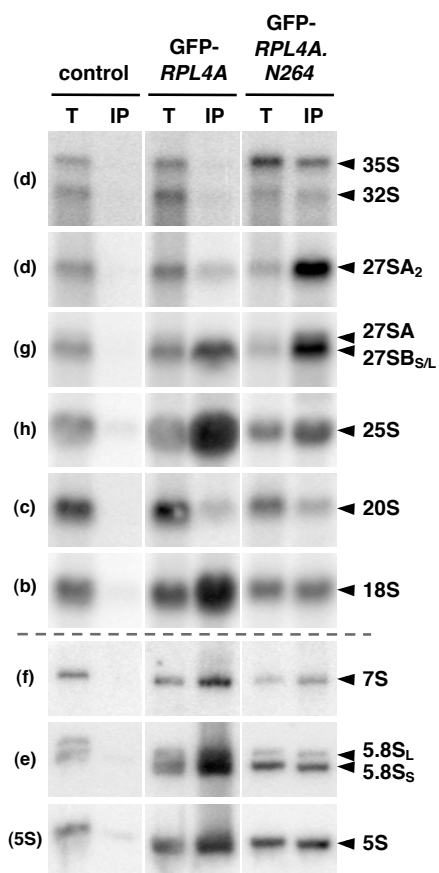

Supplement: S6 Fig — Wild-type cells containing plasmids expressing untagged Rpl4a (control) or N-terminally yEGFP-tagged Rpl4a (GFP-RPL4A) or Rpl4a.N264 (GFP-RPL4A.N264) were grown in SC-Leu medium to an OD600 of 0.8. Immunoprecipitation was carried out by incubation of cell extracts with GFP-Trap_A agarose beads. RNA was extracted from the beads and an aliquot of the total extracts. The isolated RNAs, corresponding to 1% of the total extracts (T) and 45% of the immunoprecipitates (IP), were separated on 1.2% agarose gels containing 6% formaldehyde (upper panel) or on 7% polyacrylamide gels containing 8 M urea (lower panel), transferred onto a nylon membrane, and hybridized with the indicated probes (see S1 Fig for their location within the 35S pre-rRNA). (PDF) [file pgen.1005565.s008.pdf]

Figure S7

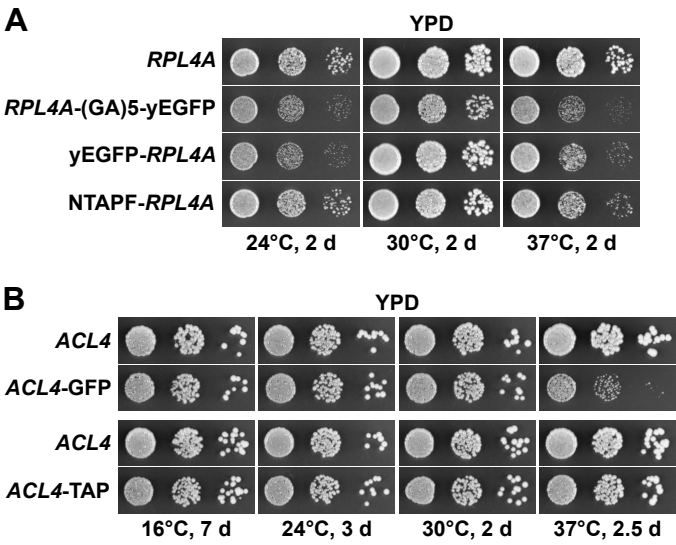

Supplement: S7 Fig — A, Growth phenotype of cells expressing GFP- and TAP-tagged Rpl4a variants. YCplac111-based plasmids harbouring the genes encoding, under the transcriptional control of the cognate promoter, C-terminally yEGFP-tagged Rpl4a (RPL4A-(GA)5-yEGFP), N-terminally yEGFP-tagged Rpl4a (yEGFP-RPL4A), or N-terminally TAP/Flag-tagged Rpl4a (NTAPF-RPL4A) were transformed into the RPL4 shuffle strain YBP15. After plasmid shuffling on 5-FOA-containing plates, cells were restreaked on YPD plates and then spotted in 10-fold serial dilution steps onto YPD plates, which were incubated for the indicated times at 24°C, 30°C, and 37°C. B, Growth phenotype of cells expressing Acl4-GFP and Acl4-TAP from the genomic locus. Cells of the ACL4-GFP and ACL4-TAP strains, as well as cells of isogenic wild-type strains (ACL4), originating from spore clones of the same tetrads as the ACL4-GFP and ACL4-TAP strains, were spotted in 10-fold serial dilution steps onto YPD plates, which were incubated for the indicated times at 16°C, 24°C, 30°C, and 37°C. (PDF) [file pgen.1005565.s009.pdf]

Figure S8

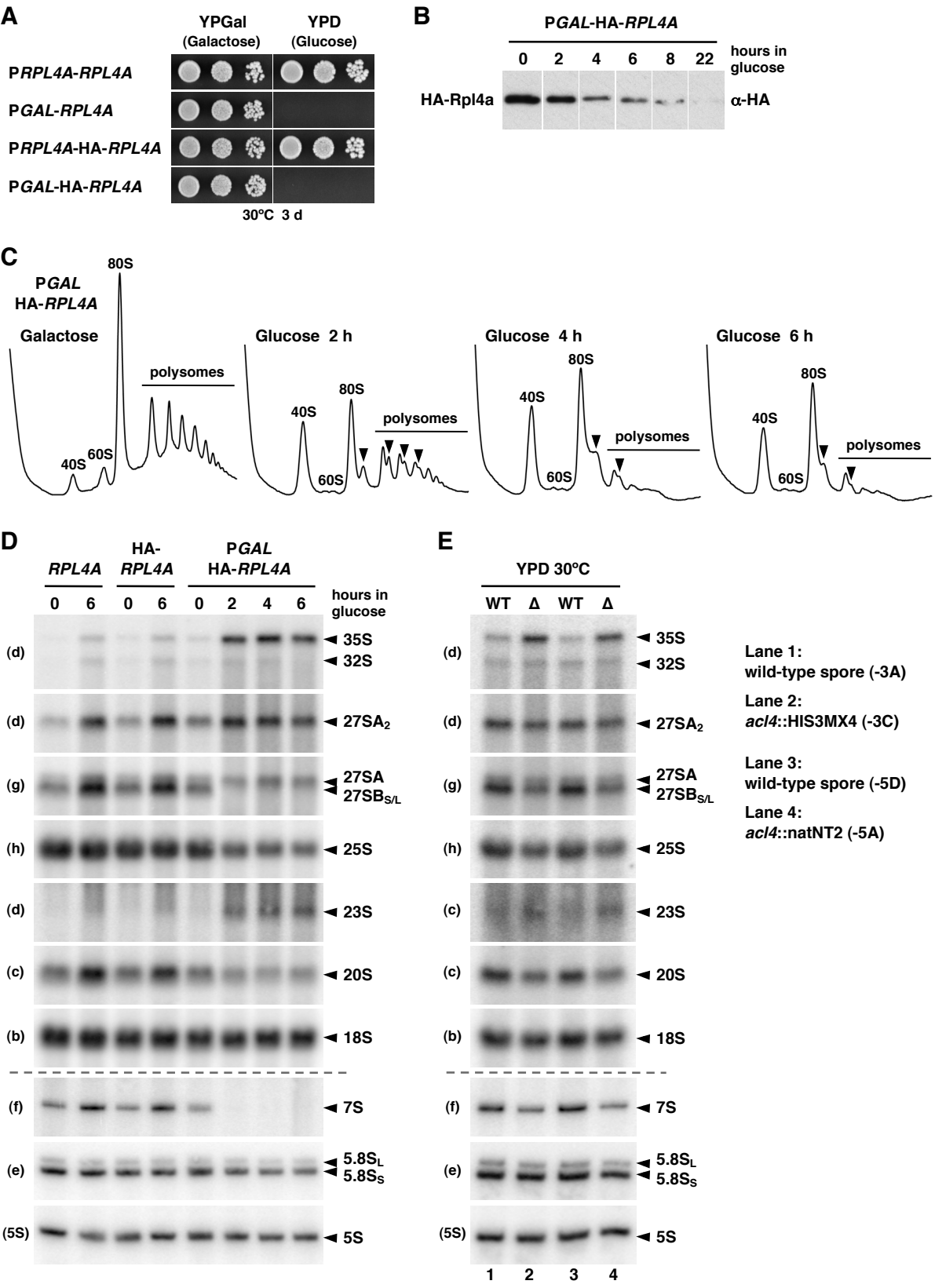

Supplement: S8 Fig — A, Genetic depletion of Rpl4 abolishes growth of yeast cells. The RPL4 shuffle strain YBP15 was transformed with plasmids expressing untagged or N-terminally 2xHA-tagged Rpl4 from either the cognate RPL4A promoter or the GAL1-10 promoter. After plasmid shuffling on SGal plates containing 5-FOA, cells were restreaked on YPGal plates and then spotted in 10-fold serial dilution steps onto YPGal and YPD plates, which were incubated for 3 d at 30°C. B, Time-course of Rpl4a depletion. Cells expressing N-terminally 2xHA-tagged Rpl4a from the galactose-inducible GAL1-10 promoter, as the sole cellular Rpl4 source, were first grown at 30°C in YPGal medium and then shifted to YPD medium. Cell extracts were prepared from samples harvested after the indicated times of growth in YPD medium (hours in glucose) and subjected to Western blot analysis using an anti-HA antibody. Note that the lanes of the different time points have been cut out from a Western blot image containing additional time points. C, Depletion of Rpl4 results in a reduced production of 60S subunits. The above strain (PGAL-HA-RPL4A) was grown at 30°C in YPGal medium and then shifted to YPD medium. Cell extracts were prepared, from cells grown in YPGal medium or from samples harvested at the indicated times of growth in YPD medium, under polysome-preserving conditions. Eight A260 units were resolved in 10–50% sucrose gradients and the absorption profiles were recorded by continuous monitoring at A254. Sedimentation is from left to right. The peaks of free 40S and 60S subunits, 80S free couples/monosomes, and polysomes are indicated. Half-mers are highlighted by arrowheads. D, Effects of Rpl4 depletion on steady-stated levels of pre-rRNAs and mature rRNAs. Strains expressing, as the sole Rpl4 source, untagged (RPL4A) or N-terminally 2xHA-tagged Rpl4a (HA-RPL4A) from the cognate RPL4A promoter or N-terminally 2xHA-tagged Rpl4a (PGAL-HA-RPL4A) from the GAL1-10 promoter were first grown at 30°C in YPGal medium and the [file pgen.1005565.s010.pdf]

Figure S9

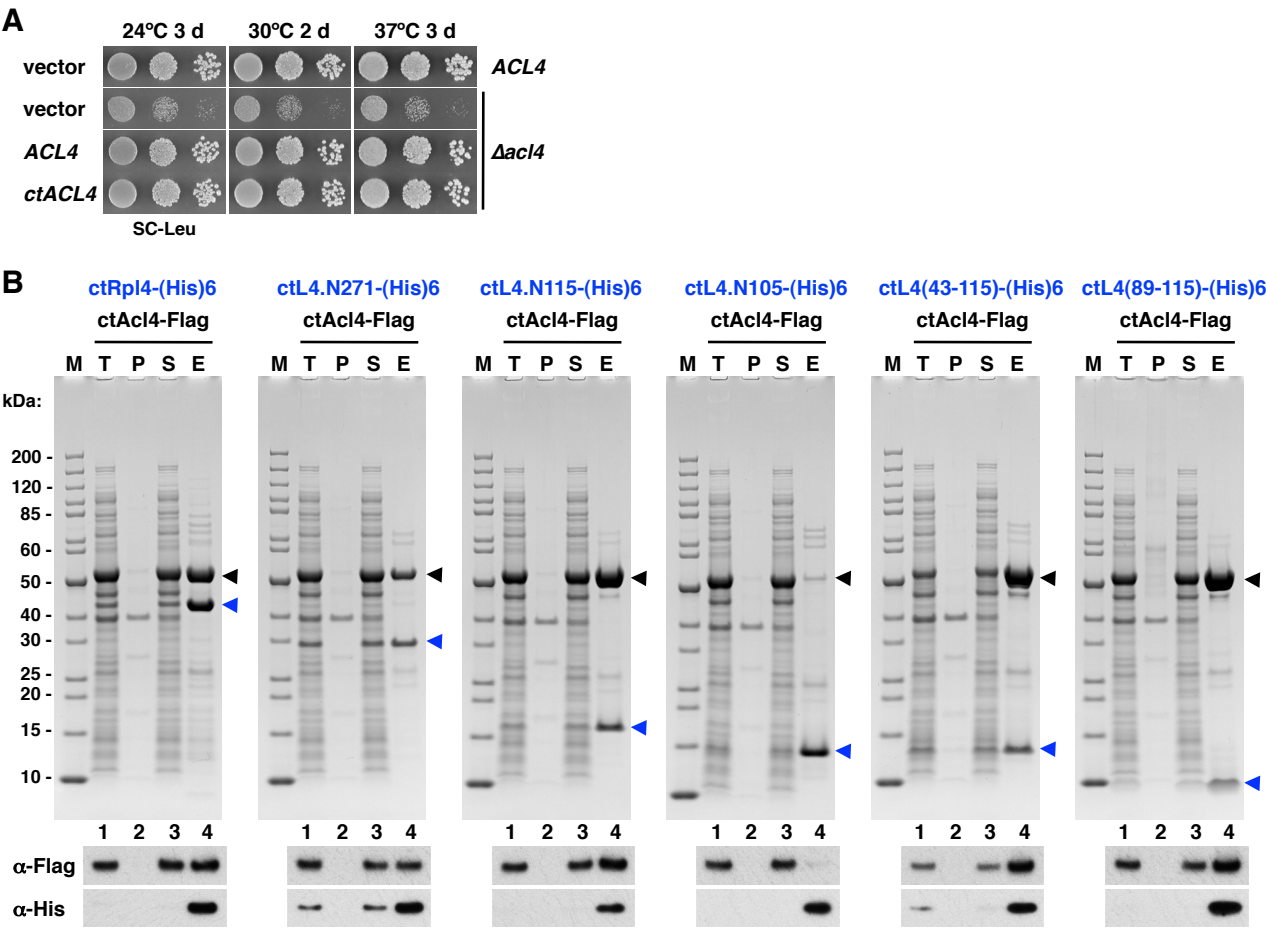

Supplement: S9 Fig — A, C. thermophilum Acl4 complements the growth defect of Δacl4 null mutant cells. The Δacl4 null mutant was transformed with YCplac111 (empty vector), YCplac111-ACL4, and pADH111-ctACL4. As a control, an isogenic wild-type strain was transformed with YCplac111. Transformants were restreaked and cells were spotted in 10-fold serial dilution steps onto SC-Leu plates, which were incubated for the indicated times at 24°C, 30°C, and 37°C. B, In vitro binding assay between ctRpl4 and ctAcl4. The indicated C-terminally (His)6-tagged ctRpl4 variants and full-length ctAcl4-Flag were co-expressed in E. coli and purified via Ni-affinity purification. Proteins were revealed by SDS-PAGE and Coomassie staining (top) or by Western blot analysis using anti-Flag (ctAcl4-Flag) and anti-His (ctRpl4-(His)6 variants) antibodies (bottom). T, total extract (lane 1); P, pellet fraction (insoluble proteins, lane 2); S, soluble extract (lane 3); E, imidazole eluate (lane 4); M, molecular weight standard. Blue arrowheads highlight the bands corresponding to the different ctRpl4-(His)6 variants used as baits for the purifications. Black arrowheads indicate the position of ctAcl4-Flag. (PDF) [file pgen.1005565.s011.pdf]

Figure S10

A

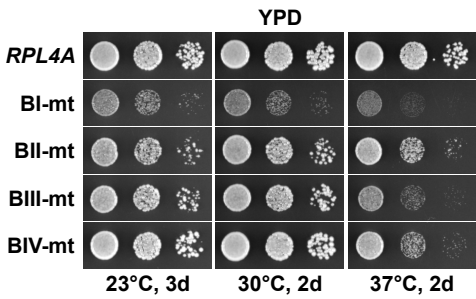

B

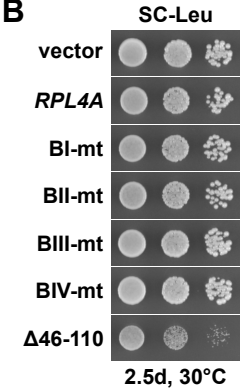

C

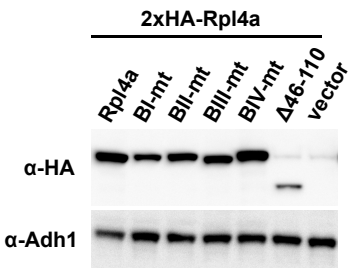

Supplement: S10 Fig — A, In vivo phenotypes of cells expressing Rpl4a variants containing non-overlapping, consecutive alanine substitutions within the C-terminal part of the long internal loop. The amino acid substitutions within each of the four alanine-block mutants (BI-mt, BII-mt, BIII-mt, and BIV-mt) are indicated in Fig 6A. YCplac111-based plasmids expressing, under the control of the cognate promoter, full-length Rpl4a or the indicated alanine-substitution mutants were transformed into the RPL4 shuffle strain YBP15. After plasmid shuffling on 5-FOA-containing plates, cells were restreaked on YPD plates and then spotted in 10-fold serial dilution steps onto YPD plates, which were incubated for the indicated times at 23°C, 30°C and 37°C. B, Expression of Rpl4a alanine-substitution variants does not confer a growth defect to wild-type cells. Empty vector (YCplac111) and plasmid-borne wild-type RPL4A or the indicated alanine-substitution mutants, expressed under the control of the cognate promoter, were transformed into the haploid wild-type strain YDK11-5A. Transformants were restreaked and cells were spotted in 10-fold serial dilution steps onto SC-Leu plates, which were incubated for 2.5 d at 30°C. C, Expression levels of Rpl4a alanine-substitution variants. Empty vector (YCplac111) and plasmid-borne, N-terminally 2xHA-tagged wild-type RPL4A or the indicated alanine-substitution mutants, expressed under the control of the cognate promoter, were transformed into the haploid wild-type strain YDK11-5A. Expression levels were assessed by subjecting whole cell lysates to SDS-PAGE and Western analysis using anti-HA and anti-Adh1 (loading control) antibodies. (PDF) [file pgen.1005565.s012.pdf]

Figure S11

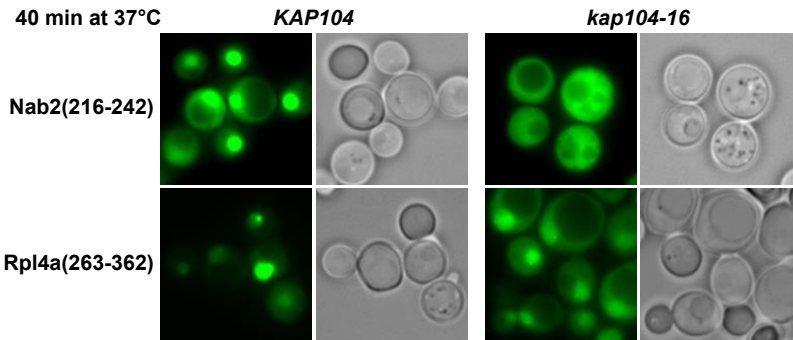

Supplement: S11 Fig — Plasmids expressing, under the transcriptional control of the ADH1 promoter, the C-terminal extension of Rpl4 (amino acids 263–362) or the PY-NLS region of Nab2 (amino acids 216–242) fused, via a (GA)5-linker, to a C-terminal 3xyEGFP were transformed into KAP104 wild-type or kap104-16 mutant cells. Transformed cells were grown at semi-permissive temperature (23°C) in SC-Leu medium and the localization was assessed by fluorescence microscopy after a shift for 40 min to the non-permissive temperature (37°C). (PDF) [file pgen.1005565.s013.pdf]

# Figure S12

**A**

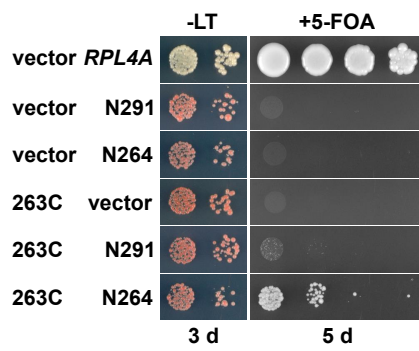

**B**

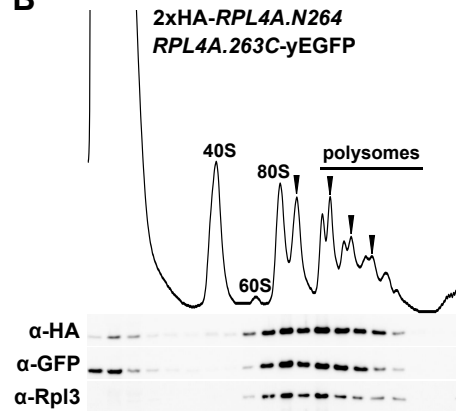

Supplement: S12 Fig — A, Separate expression of the universally conserved globular domain and the eukaryote-specific C-terminal extension results in intramolecular complementation of the Δrpl4a/Δrpl4b null mutant phenotype. The RPL4 shuffle strain YBP15 was co-transformed with empty vector or plasmids expressing full-length Rpl4a or the C-terminal extension of Rpl4a (amino acids 263–362) and empty vector or a plasmid expressing the globular domain of Rpl4a (N264 construct; amino acids 1–264). These plasmids express full-length Rpl4a and its fragments from the cognate RPL4A promoter. Transformants were restreaked on SC-Leu-Trp plates and cells were spotted in 10-fold serial dilution steps onto SC-Leu-Trp plates and 5-FOA containing (+5-FOA) plates, which were incubated for the indicated times at 30°C. B, The separately expressed globular domain and C-terminal extension get incorporated into mature 60S subunits. The RPL4 shuffle strain YBP15 was co-transformed with plasmids expressing separately the N-terminally 2xHA-tagged globular domain (N264) and the C-terminally yEGFP-tagged C-terminal extension (amino acids 263–362). After plasmid shuffling on 5-FOA containing plates, cells were grown at 30°C in YPD medium to an OD600 of around 0.8. Whole cell lysates were prepared under polysome-preserving conditions in the presence of cycloheximide and analyzed by sucrose gradient centrifugation and fractionation. Five A260 units were resolved in 10–50% sucrose gradients and the absorption profile was recorded by continuous monitoring at A254 (upper panel). Sedimentation is from left to right. The peaks of free 40S and 60S subunits, 80S free couples/monosomes, and polysomes are indicated. Half-mers are highlighted by arrowheads. Gradient fractions were subjected to Western blot analysis using anti-HA, anti-GFP, and anti-Rpl3 antibodies (lower panel). (PDF) [file pgen.1005565.s014.pdf]

**Figure S13**

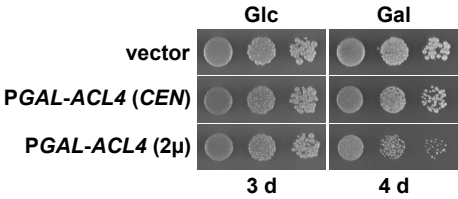

Supplement: S13 Fig — Empty vector and monocopy or multicopy plasmids expressing Acl4 under the transcriptional control of the inducible GAL1-10 promoter were transformed into the haploid wild-type strain YDK11-5A. Transformants were restreaked on SC-Leu plates and cells were then spotted in 10-fold serial dilution steps onto SC-Leu (Glucose; Glc) and SGal-Leu (Galactose; Gal) plates, which were incubated for the indicated times at 30°C. (PDF) [file pgen.1005565.s015.pdf]

**Figure S14**

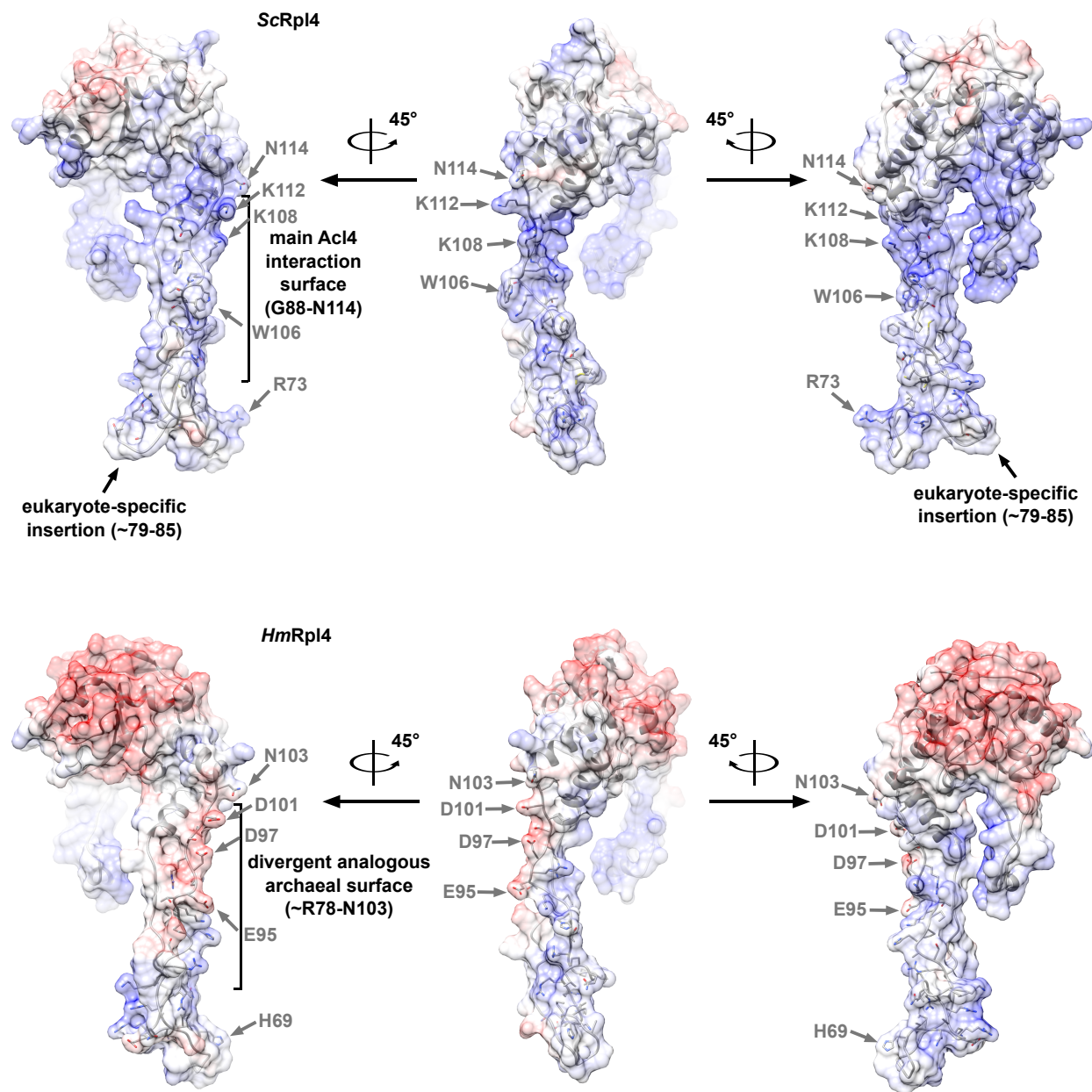

Supplement: S14 Fig — Comparison of the Acl4 binding site within the long internal loop of Rpl4 between yeast and archaea reveals that this region displays notable differences in its electrostatic surface properties. The structures of S. cerevisiae Rpl4 (amino acids 1–264) (upper panel) and full-length H. marismortui L4 (lower panel), extracted from PDB’s 4V88 and 4V9F, respectively, were generated in Chimera and are shown in dark gray as ribbon representation. Side chains of residues 88–114 of S. cerevisiae Rpl4 and residues 68–103 of H. marismortui L4 are shown and coloured by element. Coulombic surface colouring was applied to visualize, in a semi-transparent representation, the electrostatic surface properties. The following settings were used: Number of colours (default 3: red, white, blue), Range (-15 to 15 kcal/(mol·e)), Distance-dependent dielectric (true), Dielectric constant (default 4.0), and Distance from surface (default 1.4Å). The structures are shown in three different orientations. Landmark residues within the long internal loop and the main Acl4 interaction surface (amino acids 88–114), as well as the analogous archaeal surface, are indicated. (PDF) [file pgen.1005565.s016.pdf]
